# Analytical, Chemometric and Sensorial Characterization of Oloroso and Palo Cortado Sherries during Their Ageing in the *Criaderas y Solera* System

**DOI:** 10.3390/foods11244062

**Published:** 2022-12-15

**Authors:** Manuel J. Valcárcel-Muñoz, María Guerrero-Chanivet, Carmen Rodríguez-Dodero, M. de Valme García-Moreno, Dominico A. Guillén-Sánchez

**Affiliations:** 1 Bodegas Fundador S.L.U., C/San Ildefonso, n 3, 11403 Jerez de la Frontera, Spain; 2 Departamento de Química Analítica, Facultad de Ciencias, Instituto Investigación Vitivinícola y Agroalimentaria (IVAGRO), Campus Universitario de Puerto Real, Universidad de Cádiz, 11510 Puerto Real, Spain

**Keywords:** sherry wine, oxidative ageing, multiple linear regression, principal component analysis

## Abstract

Oloroso and Palo Cortado are two types of sherry wines, produced in the Sherry Wine Region in Southern Spain, known as *Marco de Jerez,* where it is aged following the traditional *Criaderas y Solera* system. All of them are aged through oxidative ageing, even though the peculiar Palo Cortado Sherry wine is also aged biologically under a veil of flor yeasts in the first stage. Total dry extract, organic acids, aldehydes, esters, higher alcohols and phenolic compounds in these sherry wines evolve during their ageing as a consequence of evaporation and/or perspiration processes, chemical reactions, extraction of compounds from oakwood and microbiological activity. Sherry wines develop their characteristic organoleptic profile during their ageing, as could be proven through their tasting sessions. According to the sherry type, some natural groupings of the wines could be observed after their principal component analysis. Furthermore, by multiple linear regression methods, an important correlation between the parameters that were analyzed and the ageing of each specific wine has been confirmed, which allowed us to establish two different models, each corresponding to the sherry type in question. Only five of the variables that were investigated were required to successfully estimate each wine’s age at over 99% confidence. This represents a rather convenient tool for wineries to monitor the ageing of these sherry wines.

## 1. Introduction

Sherry wines, which include Fino, Amontillado, Oloroso, Palo Cortado and Pedro Ximénez, are quite distinctive wines. They are produced from three different grape varieties: Palomino, Pedro Ximénez and Moscatel. Their ageing, which can be biological, oxidative or a combination of both categories, is carried out using the traditional dynamic ageing system known in Jerez as *Criaderas y Solera*. All of this, together with the particular climatic conditions in Marco de Jerez, the only region in Southern Spain where these wines are made, contributes to the unique characteristics that support their high esteem by the oenological community [1,2].

Oloroso Sherry wine is a dry wine made from Palomino grapes. These freshly harvested grapes, with more than 10.5° Baumè, are pressed to extract a must that ferments completely. After this fermentation, the young wine is racked and fortified to over 17% ABV (alcohol by volume). The young Palomino fortified wines are aged exclusively by oxidative ageing (with the absence of the veil of flor yeasts on the surface of the wine), using the *Criaderas y Solera* system that characterizes Sherry wines [3,4,5]. Oloroso Sherry wine displays an amber to mahogany color; it possesses complex and powerful aromas, with notes of nuts in the shell, such as walnuts, toasted, vegetable and balsamic aromas reminiscent of seasoned noble woods, as well as spicy notes; it is tasty on the palate, well-structured and full-bodied, with a dry and elegant finish [4,6].

Palo Cortado Sherry wine is a dry wine also made from Palomino grapes, with a very particular winemaking process that combines the first stage of the regular process to make Fino wines (biological ageing in a system of *Criaderas y Solera*) with its subsequent static oxidative ageing by the *Añadas* system, and finally a dynamic oxidative ageing using its own specific system in *Criaderas y Solera*. Thus, after its fermentation, the young wine is fortified by adding wine alcohol at a strength of over 15% ABV to start off the Fino ageing-exclusive process [7]. However, during this stage, not all the casks follow the same evolution, such that in certain cases, the wines aged in them exhibit unusual organoleptic characteristics of rarities, which means that they cannot continue their ageing process as Fino wines. These particular organoleptic characteristics make them special and highly appreciated for the elaboration of other types of wine. As they are identified during regular inspections by the winery oenologists, these wines are removed from their casks. Once they have been identified and removed from their casks, they are fortified with wine alcohol up to 17% ABV, which is the minimum strength required for the elaboration of Palo Cortado [8]. Then, a period of oxidative ageing by the *Añadas* system takes place, during which periodic tastings are also carried out to verify that the special organoleptic characteristics for which they were selected persist. Only those wines that maintain these unique organoleptic characteristics will be used to replenish younger *Criaderas* following the dynamic oxidative ageing process for Palo Cortado Sherry wines. This type of sherry wine exhibits a color between chestnut and mahogany and a wide variety of nuances in its aroma, where the characteristic notes of Amontillado and Oloroso Sherry wines combine harmoniously with other citric and lactic notes. It is deep and voluminous in the palate with a long and pleasant finish where the aromatic elegance of Amontillado Sherry wine and the full body on the palate of Oloroso Sherry wine are combined [9].

Both types of wines, Oloroso and Palo Cortado, are aged in 500 to 600 L American oak casks, which are arranged at different heights and/or scales according to the age of the wine in them. Each scale or ageing level is known as *Criadera*, except for the lowest one and last, which is known as *Solera*. This scale is also the oldest scale. The sherry wine intended for consumption is extracted from the Solera level. Periodic wine transfers are carried out from one cask to another: a part of the sherry wine contained in each of the casks that conform to the same ageing scale is removed (*saca*), and then, the cask is replenished (*rocío*) with sherry wine extracted from the next older scale [10,11,12]. This ageing system can, to some extent, reduce any deviations in the homogeneity of the sensorial and chemical–physical characteristics of these sherry wines in the Solera as years pass by.

The casks used for wine ageing are essential elements of the process, since they contribute with numerous compounds that are involved in certain chemical reactions with a significant influence on the wines and since they modify their organoleptic properties while considerably improving them [13,14,15]. Wood, above all, contributes with phenolic compounds [16], such as phenolic aldehydes derived from cinnamic acid (coniferaldehyde and sinapaldehyde) and benzoic acid (such as vanillin and syringaldehyde) [16,17]. Other remarkable compounds that are transferred from the wood include furfurals and their derivatives, such as 5-hydroxymethylfurfural or 5-methylfurfural [17]. Finally, pentoses and polysaccharides (from hemicellulose) [16,17], alcohols, fatty acids and inorganic substances, among others [17], are some of the additional substances that enrich these wines and provide them with their quality.

This work intends to determine the physicochemical and organoleptic characterization of two different Soleras of Sherry wines—Oloroso and Palo Cortado—aged through oxidative systems. More specifically, this research intends to determine the characteristics of these wines as well as the correspondence of any of the trends exhibited by each of the parameters under study with the average age of the wines from different ageing categories or scales. Thus, by employing chemometric classification techniques, suitable models have been developed to differentiate both between wines and according to their average age. We expect these models to be a rather useful tool for the industry as well as for routine quality control procedures.

## 2. Materials and Methods

### 2.1. The Samples

The Oloroso and Palo Cortado Sherry wines, which are the subject of this study, the casks used in the study, the wine alcohol used for the fortifications, and the ageing cellar facilities where this study was carried out were all made available by Bodegas Fundador, S.L.U. (Jerez de la Frontera, Cadiz, Spain). All the wines in this study complied with the specifications set out in the specifications for the Jerez–Xérès–Sherry Protected Designation of Origin (P.D.O.) [18,19].

For this research, *Quercus alba* cask, of 500/600 L capacity, and medium toast were selected. All these casks were part of industrial *Añadas* and *Criaderas y Solera* systems to Bodegas Fundador S.L.U. All these casks were supplied by cooperages from Jerez de la Frontera, Spain.

**Figure 1:**
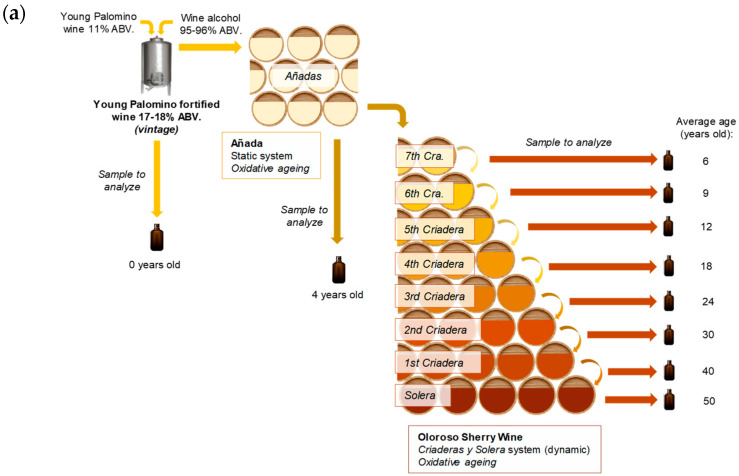

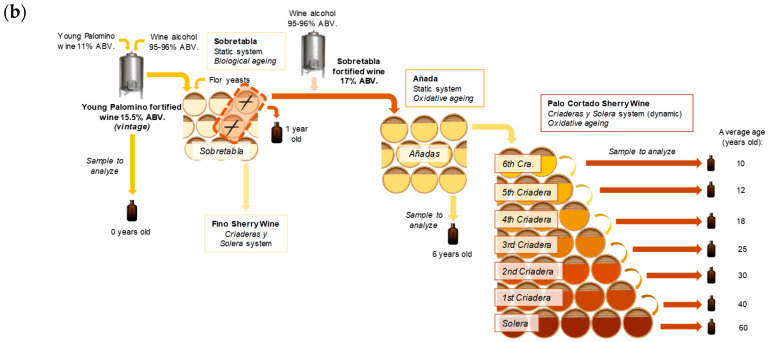
Ageing and sampling diagram of the sherry wines: (**a**) Oloroso Sherry; (**b**) Palo Cortado Sherry.

All the pooled samples were taken throughout the 4 years of the study (4 samples from each type of wine and ageing stage, *n* = 4) and were physicochemical characterized. All the analyses were performed in triplicate on each sample. During the 4 years of the study, the wines were annually checked for any possible defects. The Oloroso and Palo Cortado wines were physicochemically characterized and sensorially evaluated using the samples that are listed below. Figure 1 shows the diagram of the winemaking process for each of the two types of wine studied.

Oloroso Sherry wine processing: every year, 150,000 L of young wine was fortified at 17–18% ABV and stored in 5 industrial 30,000 L stainless steel tanks to allow for the replenishment of the Oloroso Sherry wine *Añadas*. In this *Añadas* system, wines are aged for 4 years under static oxidative ageing. Likewise, each year, fifty 500 L casks of 4-year old Oloroso Sherry *Añadas* wine are used to replenish the youngest ageing scale in the Oloroso Sherry wine *Criaderas y Solera* system. A system consisting of seven *Criaderas* (Cra.) and one *Solera* with fifty 500 L casks per scale was set up, and the corresponding *sacas* and *rocíos* were conducted as appropriate. The average age of the different scales can be seen in Figure 1. The Oloroso Sherry *Añadas* wine was 4 years old. Regarding the *Criaderas y Solera* system, the 7th Cra. was an average of 6 years old; the 6th Cra., 9 years; the 5th Cra., 12 years; the 4th Cra., 18 years; the 3rd Cra., 24 years; the 2nd Cra., 30 years; the 1st Cra., 40 years; while the Solera was 50 years old.

Palo Cortado Sherry wine processing: each year, 150,000 L of young wine fortified at 15.5% ABV was stored in five 30,000 L stainless steel tanks for the *rocío* of the *Sobretabla*. According to this system, the wines are subjected to 10–12 months of static biological ageing. In addition, each year, twenty five 500 L casks of wine were selected by the cellar oenologists to produce Palo Cortado in the *Sobretabla* and to replenish the Palo Cortado Sherry wine *Añadas*. According to this *Añadas* system, the *Añadas* wines are aged for 5 years following a static oxidative process. Likewise, each year, twenty-five 500 L casks of 6-year-old Palo Cortado Sherry *Añadas* wine are used to replenish the youngest ageing scale of the Palo Cortado *Criaderas y Solera* system. This system consisted of six Criaderas and one Solera, each one of them comprising twenty-five 500 L casks, all of which underwent the traditional *sacas* and *rocíos* procedures. The Palo Cortado Sherry wine in the *Sobretabla* was 1 year old, and the Palo Cortado Sherry wine in *Añadas* was 6 years old. The 6th Criadera (6th scale) in a *Criaderas y Solera* system is on average 10 years old; the 5th Cra. is 12 years old; the 4th Cra. is 18 years old; the 3rd Cra. is 25 years old; the 2nd Cra. is 30 years old; the 1st Cra. is 40 years old; and the Solera has an average age of 60 years.

The study spanned over four years, with all the wines of different types and ages being sampled once per year. The procedure was adapted to the characteristics of the ageing systems under study, so that each type of wine was sampled annually. During the 4 years of the study, and before sampling, all the wines were tasted to verify that they did not present organoleptic defects. The two types of young fortified wines were sampled in January. For this, a composite 5 L sample was made up by one liter of wine from each of the 5 tanks containing each type of wine. The remaining wines were sampled in the months of May. For the Oloroso Sherry wine Añadas, a 5 L pooled sample was prepared using 100 mL from each of the 50 casks that constituted the Añadas. Two 5 L samples were produced for Palo Cortado, the first one by adding 200 mL from each of the 25 wines that had been selected in the Sobretabla by the winemakers to produce Palo Cortado, and the second sample of Palo Cortado Sherry wines in Añadas that was conformed by 200 mL from each of the 25 casks that made up the total of each type of wine, which resulted in a 5 L sample for each of them. From each of the two Criaderas y Solera systems under study, 200 mL was taken from each of the 25 casks that contained each type of wine and age, to obtain a final 5 L sample from each of them.

#### Environmental Conditions of the Cellar and Effects of Volume Loss by Evaporation and/or Transpiration during Ageing

The average conditions of humidity and temperature of the cellar are shown in Table 1.

The result mainly of the concentration due to the transpiration of water molecules through the casks’ wood pores is traditionally known as *merma* (evaporation and/or perspiration loss) [7,20].

The average annual *merma* volume in the *Añadas* and *Criaderas y Solera* systems over the 4 years of the study was approximately 3.0%. However, these volume losses are not detectable in the casks, since they are annually replenished with the next older wine, and this partly compensates for the losses. Table 2 shows the increasing concentration of compounds that result from this effect relative to the average age of the wine and for all the periods studied.

### 2.2. Parameters of Oenological Control

The measurement of the alcoholic strength (%ABV) was carried out by means of distillation of the wine and subsequent measurement of the density of the distillate [21] using a DMA-5000 digital densimeter (Anton Paar, Ashland, OR, USA).

For the direct measurement of the density of the samples, a DMA-5000 digital densimeter (Anton Paar, Ashland, OR, USA) was used.

The pH of the samples was measured by means of a Basic 20 pH meter (Crison Instruments SA, Barcelona, Spain). The total acidity was measured by potentiometric titrations at pH 7 [22]; the results were expressed as gram of tartaric acid per liter.

An AA3 HR Autoanalyzer segmented flow analyzer (Seal Analytical, Norderstedt Stadt, Germany) [23] was used to determine the volatile acidity. The results were expressed as gram of acetic acid per liter.

The official standard enzymatic method [24], the Ripper method [25], the gravimetric method [26], the officially established procedure [27], and the gravimetric method according to the barium sulfate precipitation [28] were used to determine the glycerol content in g/L, the total sulfur content in mg/L, the total dry extract in g/L, the reducing substances in g/L, and the amount of sulfates, expressed in gram of potassium sulfate per liter. The sugar-free extract, expressed in g/L, was obtained by Equation (1) [26].

(1) $$\mathrm{Sugar}-\mathrm{free}\;\mathrm{extract}\;\left(\frac{g}{L}\right)=\;\mathrm{Total}\;\mathrm{dry}\;\mathrm{extract}\;\left(\frac{g}{L}\right)-\mathrm{Reducing}\;\mathrm{substances}\;\left(\frac{g}{L}\right),$$

The concentration of potassium [29], calcium [30], iron [31] and copper [32] in mg/L, was obtained by atomic absorption spectroscopy, using a PinAAcle 900F (Perkin Elmer, Boston, MA, USA) equipped with WinLab32 AA software (Perkin Elmer, Boston, MA, USA).

All the analyses were conducted in triplicate.

### 2.3. Organic Acids

Citric, lactic, malic, succinic and tartaric acids were determined by ion chromatography using a 930 Compact IC Flex (Metrohm, Madrid, Spain), fitted with a 250 × 7.8 mm (i.d.) and 9 µm particle size Metrosep Organic Acids column [7]. MagicNet 3.3 (Metrohm, Madrid, Spain) was the software application used for data acquisition and processing. The compounds were identified by comparing retention times and standards. All the calibration standards were supplied by Sigma-Aldrich (Saint Louis, MO, USA). The results were expressed in mg/L. The standards and samples were injected in triplicate.

### 2.4. Aldehydes, Methanol, Higher Alcohols and Esters

Methanol, acetaldehyde, acetaldehyde-diethylacetal, esters (ethyl acetate, ethyl decanoate, ethyl dodecanoate, ethyl hexadecanoate, ethyl hexanoate, ethyl lactate, ethyl octanoate, diethyl succinate and ethyl tetradecanoate) and higher alcohols (hexanol, isobutanol, 2-methyl-1-butanol, 3-methyl-1-butanol, 2-phenyl ethanol and n-propanol) were determined by GC-FID. These analyses were performed on an Agilent 7890B Gas Chromatograph (Agilent Technologies, Santa Clara, CA, USA) coupled to a flame ionization detector. The methodology used was described in previous studies by our research group [7,33]. The samples were directly injected in triplicate. The results were expressed in mg/L.

### 2.5. Folin–Ciocalteau Index

The Folin–Ciocalteu index (CFI) was used to determine the total phenolic content in milligrams of gallic acid equivalent per liter [34]. Folin–Ciocalteau reagent (Merck, Darmstadt, Germany), anhydrous sodium carbonate (Merck, Darmstadt, Germany) and ultra-pure water (EMD Millipore, Bedford, MA, USA) were used as reactive. To determine the absorbance at 750 nm, a Lambda 25 spectrophotometer (Perkin Elmer, Boston, MA, USA) was used, using glass cells with a light path of 10 mm. The results were expressed as milligram of gallic acid per liter. All samples were analyzed in triplicate.

### 2.6. Furfurals and Phenolic Compounds

Fifteen phenolic compounds, gallic acid, caffeic acid, cis-p-coutaric acid, fertaric acid, ferulic acid, p-coumaric acid, p-hydroxybenzoic acid, protocatechuic acid, syringic acid, trans-caftaric acid, trans-p-coutaric acid, vanillic acid, p-hydroxybenzaldehyde, syringaldehyde and vanillin) and two furfurals (furfural and 5-hydroxymethylfurfural) were quantified by UPLC [33,35].

The UPLC system used was a Waters Acquity UPLC system (Waters Corporation, Milford, MA, USA) equipped with an Acquity UPLC C18 BEH column, 100 × 2.1 mm i.d. and 1.7 µm particle size and a PDA detector. Samples and standards were filtered through 0.22 µm pore size nylon membranes and injected in triplicate. The retention time and UV–Vis spectra of the sample and those of the standards were used to identify the compounds analyzed by comparison The results were expressed in mg/L.

### 2.7. Color Measurements

For the determination of the color of the samples in absorbance units (U.A.), the official method established by the International Organization of Vine and Wine (OIV) [36] was used. The absorbance at 470 nm was measured with a Lambda 25 spectrophotometer (Perkin Elmer, Boston, MA, USA). This wavelength is related to the colors that wines acquire as a result of ageing: yellow tones, old gold, topaz, amber or mahogany, among others. All measurements were performed in triplicate.

### 2.8. Tasting Sessions

The tasting sessions were conducted in a room designed to facilitate the isolation and concentration of the tasters, who carried out their tasting individually and at an ambient temperature of 20 °C [37]. Seven tasters, all of them members of the winemaker company’s tasting panel and with over 10 years of experience in sherry wine tasting, took part in the study. Furthermore, 3 of them are members of the official tasting panel for the Protected Designation of Origin (P.D.O.) Jerez–Xérès–Sherry.

Oloroso and Palo Cortado Sherry wines with two different average ages (12 years and over 30 years) were sensorially evaluated. Specifically, the evaluated wines were the following: Oloroso and Palo Cortado Sherry wines with 12 years of age (5th Criadera), since the Jerez–Xérès–Sherry Specifications allow for certification of a wine with a certified age of 12 years, and a blend consisting of 50% 1st Criadera (40 years) and 50% 2nd Criadera (30 years), which results in an average age of over 30 years, since the P.D.O. Jerez–Xérès–Sherry Specifications allow for the certification of a wine with an average age of more than 30 years (VORS—Very Old Rare Sherry) [18,19].

Then, 50 mL of each sample was served into black wine tasting glasses [38] and covered with a glass lid for at least ten minutes in order to stabilize their headspace before they were tasted. Nose and mouth sensory data were registered.

The criteria of the tasting panel of the D.O.P. Jerez–Xérès–Sherry were used to select the descriptors used in the organoleptic analysis of the samples. The definitions of these descriptors, as well as the olfactory–gustatory patterns for the maximum intensity of each one are shown in Table 3. A numerical scale of 9-point intervals was used [39]. Selected wines from the Solera category (listed in Table 3) were used as the maximum intensity standard (value: 9), and a young vintage wine of the Palomino variety (11% ABV) was used as the minimum intensity standard (value: 1).

### 2.9. Statistical Analysis

For ANOVA, Fisher’s least significant difference test, principal component analysis (PCA), and multiple linear regression analysis (MLRA), the Statgraphics 19 software package (Statgraphics Technologies, Inc., The Plains, VA, USA) was used. The other statistical parameters were calculated using Microsoft Excel 2016 (Microsoft Corp., Redmond, WA, USA).

The statistical analysis of the sensory data included analyses of variance where the sample, the judge and a factor analysis were the variance factors. The statistical software Statistica 8.0 (StatSoft Inc., Tulsa, OK, USA) was used for these analyses. A spider-web diagram was generated by means of Microsoft Excel 2016 (Microsoft Corp., Redmond, WA, USA).

## 3. Results and Discussion

### 3.1. Parameters of Oenological Control in Oloroso and Palo Cortado Sherry wines

#### 3.1.1. Alcoholic Strength

Significant differences between the alcoholic graduations of Oloroso Sherry wine and Palo Cortado Sherry wine were registered for all the scales studied (Table 4).

An increase in the alcohol content in both wines was observed with age. This was the result mainly of the concentration due to the merma. This effect widely exceeds the decreases in ethanol that occur in wine due to the chemical reactions in which it intervenes to reach its equilibrium (oxidation, esterification, etc.), giving rise to compounds such as acetaldehydes, acetals, ethyl acetate, acetic acid, etc.

#### 3.1.2. pH, Total Acidity and Volatile Acidity

An age-dependent pH increase in Oloroso and Palo Cortado Sherry wines *Criaderas y Solera* systems could be observed (Table 4). The Oloroso Sherry wines showed pH values between 3.30 and 3.45, while the Palo Cortado Sherry wines were slightly more acidic and reached a pH value of 3.24 in the Solera. This pH evolution can be explained by a growing proportion of certain organic and inorganic acids that come either from the grapes or have been added as part of certain prefermentative practices such as plastering [40,41,42,43]. These acids may also be generated by the alcoholic and/or malolactic fermentation processes. However, both of these processes also cause an important concentration of calcium and potassium in the form of salts such as calcium tartrate, calcium sulfate and potassium bitartrate that make some acids become insoluble, since they are supersaturated in the medium. Consequently, due to the significant precipitation of potassium bitartrate, calcium tartrate and calcium sulfate that takes place in the casks during their first winter of ageing, the total acidity of both the Oloroso and Palo Cortado Sherry wines decrease with respect to that of the young Palomino fortified wine.

The increase in organic (except for tartaric acid, which decreases by precipitation of its salts) and inorganic acids during ageing causes an increase in total acidity, which is not reflected in a decrease in pH values, since there is also an increase in potassium, which is supersaturated in the medium and is responsible for the pH increasing only slightly. This certain buffering effect of potassium is very important, since in very long-aging wines such as those studied, if it did not exist, there would be very acid and astringent products that would be rejected by consumers.

The two wines studied exhibited similar behaviors in terms of total acidity. This parameter increases with ageing (with the exception of the *Añadas* ageing stage, as above mentioned). This may be due to the oxidation of the ethanol in the medium, which increases the acetic acid content [33], the concentration of organic acids due to the *merma* and the transferring of acidic compounds from the wood [17]. Acidity increased despite the reduction in the tartaric acid content that takes place with ageing as tartaric salts precipitate.

Volatile acidity increases in all the wines as a consequence of the alcohol oxidation that takes place in the medium [33], the acetic acid contributions from the wood [17], and the effects attributable to the *merma* phenomenon.

While pH did not present any clear trends according to the ANOVA results, significant differences were observed between the total acidity values of the different ageing scales of all the wines under study, as well as between their volatile acidity values.

#### 3.1.3. Total Sulfur Dioxide

The initial sulfur dioxide values in the young wines for the initial sulfite content in the musts between 80 and110 mg/L at fermentation were as expected. In addition, the two sherry wines studied presented a decrease in their total sulfur content with ageing that went down to practically 0 mg/L in the Solera (Table 4). Sulfur oxidized into sulfate and decreased, as no new sulfur was added after the fortification. Noticeable differences were observed between the total sulfur values corresponding to the different ageing scales in all the wines under study.

#### 3.1.4. Sulfates

Both the Oloroso and the Palo Cortado Sherry wines studied exhibited an increment in sulfates with ageing time (Table 4). A marginal part of such a trend could be related to the presence of sulfur, while the effect of *merma* seems to be mainly responsible for this sulfate concentration increment (Table 2). However, a greater increment in sulfate concentration was expected versus that measured, which could be due to certain insolubilizations, and mainly to that of calcium sulfate [44].

No significant differences were observed between the sulfate content in the first ageing scales of the two wines studied, even if statistically significant differences could be observed between the different scales as ageing time increased.

Its content in the young fortified wine depends on that of the grapes themselves and on the sulfates added when plastering [43].

#### 3.1.5. Calcium and Potassium

A decrease in calcium and potassium content was initially observed in the early ageing stages of the two types of wine studied (Table 4), while the older wines exhibited greater contents as a result of the *merma* (Table 2). Such decreases are due to the precipitation of organic and inorganic salts during ageing. The potassium–tartaric acid balance depends on the alcohol content in the wine, although it is always supersaturated in the medium, which is why precipitations of potassium bitartrate take place when the cellar temperature goes down. In the case of calcium, the precipitations that occur involve calcium salts of tartrates and sulfates, as well as oxalate in certain old age wines [45], which present higher concentrations than the usually attributable to the *merma* effect (Table 2). Most of the wines studied exhibited significant differences between potassium and calcium content as a function of ageing.

#### 3.1.6. Glycerol

Glycerol levels rose over the ageing time for all the sherry wines studied (Table 4). The concentration of this compound is related to transpiration and/or evaporation due to the *merma* phenomenon (Table 2) that takes place over the ageing period.

Most of the glycerol content in wine is a by-product of alcoholic fermentation processes [46]. The highest glycerol content levels are observed in Palo Cortado Sherry wine where it reaches values of 16.8 g/L in the Solera, with an average ageing period of 60 years (despite its concentration drop over the 10–12 months of biological ageing). This compound is involved in the metabolism of the yeasts of the veil of flor [7] during the biological ageing stage of this wine, where it is consumed as a source of carbon for cell regeneration; hence, its content declines during this stage. Once this sherry wine enters oxidative ageing, this compound does not disappear, but increases its concentration as an effect of the *merma*. The glycerol content in Oloroso Sherry wines is similar, although slightly lower than that in Palo Cortado Sherry wines, where it reaches 16.2 g/L in the Solera with 50 years of average age. The difference between the concentrations in the two wines could be due to the fact that the Solera of Palo Cortado Sherry wine is on average 10 years older than that of Oloroso. Thus, given that this compound increases its concentration with the *merma*, the older the wine, the higher the glycerol concentration that can be found.

Significant differences in glycerol content were observed according to the ageing of all the wines in the study.

#### 3.1.7. Total Dry Extract, Density, Reducing Substances, Sugar-Free Extract

The density of the Oloroso and Palo Cortado Sherry wines remains practically unchanged during their ageing, which is why not all their measurements present relevant differences. A slight increment of this value can be observed in the Oloroso Sherry wine, while the Palo Cortado Sherry wine shows a slight decrease, although it remains practically the same for many of the scales. The fact that there are no changes in the density values during the ageing process in either of these dry wines seems to be logical, given that the amount of soluble solids increases in the wine as a result, on the one hand, of the *merma* that increases their density, and, on the other, their alcohol content that decreases it.

The values for total dry extract, along with those of reducing substances and sugar-free extract increased over the ageing period in all the wines studied (Table 4).

The total dry extract in the two types of wine studied increased with ageing due to the contribution of wood and the *merma* effect, which affect all the compounds responsible for the quantification of this extract: glycerol, acids, phenolic compounds and reducing substances (hexoses, pentoses, and polysaccharides extracted from the oakwood).

During the oxidative ageing stage, the two wines studied increased their concentration of “reducing substances” owing both to the effect of the *merma* and to the pentoses, hexoses and polysaccharides from hemi-cellulose transferred from the wood over this period [16,17]. Sugars and polysaccharides are the main components included in the quantification of this parameter. The measured levels were very similar in Oloroso and Palo Cortado Sherry wines. In the case of Palo Cortado Sherry wine, some traces of sugars, mainly fructose, may remain in the young fortified wine after its fermentation. These residual sugars (1–2 g/L) are used by the veil of flor yeasts during the biological ageing stage (*Sobretabla*) as their primary source of carbon for cell multiplication. As a consequence of this, their concentration is appreciably low when the level of reducing substances in Palo Cortado Sherry wine are compared against those in Oloroso Sherry wine, where no veil of flor is developed. This can also be noticed in the controlled wines that conform the *Añadas* for each type of wine.

The sugar-free extract does not include any sugary or polysaccharide substance. It is a measurement of all those non-volatile substances at 110 °C that increase their concentration over the ageing years. The origin of these substances can be the grapes themselves, but they may also be generated by either alcoholic or malolactic fermentation processes in the wine, or by the contributions from the wooden casks where the ageing takes place, or as a result of the *merma* or even by other factors. An increase in this analytical parameter was observed in the two types of wines undergoing oxidative ageing. Thus, the Solera of the Oloroso Sherry wine revealed a higher sugar-free extract level at 32.07 g/L, compared to the 30.97 g/L that was recorded for the Palo Cortado Sherry wine Solera.

Significant differences were observed between all the total dry extracts, reducing substances and sugar-free extracts corresponding to each ageing scale of all the wines under study.

### 3.2. Organic Acids of Oloroso and Palo Cortado Sherry Wines

The contents of organic acids (citric, lactic, malic, succinic and tartaric acids) in wines depend on the amount of these substances that are already present in the grape itself, as well as on the activity of the yeasts and bacteria, which in their metabolic pathways, may increase or decrease their organic acid concentrations [46]. With the exception of tartaric acid, whose organic acid concentration decreases during the wine ageing due to the insolubilizations of potassium and calcium salts [7,44], all of them tend to present a growing trend during oxidative ageing (Table 5) as a result of the *merma*.

Significant differences can also be observed in the amount of total organic acids present in the wines from all the ageing scales of the different wines, except for the proportion of malic acid in the Palo Cortado Sherry wine Solera. This exception is explained by its peculiar initial biological ageing that involves malolactic fermentation.

The grapes are the main source of citric acid for the two types of wine under study, and therefore, they reach the wine through the crushing of the grapes. An increment of this acid over the ageing time can be observed in these wines as a result of the *merma*, both during the *Añadas* static oxidative ageing and over the dynamic ageing period in *Criaderas y Solera*. The biological ageing that Palo Cortado undergoes is an exception to this behavior, since during its initial biological ageing stage (*Sobretabla*), when winemakers identify those casks that contain the singular product that can give rise to this unique type of wine, the metabolic activity by the yeasts [47] and by the lactic acid bacteria [7] make the concentration of citric acid reduce.

Malic acid follows a similar behavior to citric acid, and it also comes from the grapes. All the wines in the study increased their concentration due to the *merma* effect when aged under oxidative conditions. Only in the biological ageing stage of the Palo Cortado Sherry wine did its content decrease as a consequence of the malolactic fermentation that is carried out by lactic acid bacteria. The same behavior was demonstrated by a previous study on two other sherry wines as Fino or Amontillado [7].

Succinic acid is produced by the metabolic activity of yeasts during the fermentation of the must [46]. Similarly, during the biological ageing stage of Palo Cortado Sherry wine, a decrease in the content of this acid can be observed as a result of the metabolism of the veil of flor yeasts that assimilate it [47]. All the wines studied increased their concentration of this acid due to the *merma* phenomenon (Table 2) as they aged in their oxidative systems.

Lactic acid is present in young wines in small quantities, mainly due to the metabolic activity of yeasts that produce it from sugars during the alcoholic fermentation [46]. In the case of Palo Cortado Sherry wine, during its biological ageing stage (*Sobretabla*), a significant increment of lactic acid can be observed. It comes from the activity of lactic acid bacteria (malolactic), which after the concentration effect of the *merma* in older wines, allows for this acid to be the predominant one in this type of wine.

In the case of Palo Cortado Sherry wine, the selected casks during their biological ageing stage (*Sobretabla*) contained higher concentrations of lactic acid in comparison with those that continued the process to produce Fino Sherry wine, even though their total acidities were similar [7]. This may be due to a more intense metabolic activity by the lactic bacteria (malolactic, etc.) versus that registered for those casks that were finally assigned to the ageing of Fino Sherry wines [8]. Due to this more intense metabolic activity, the yeasts in the veil of flor in the Palo Cortado Sherry casks do not have the capacity to assimilate part of the lactic acid formed.

Tartaric acid has several origins; a large part comes from the grape itself, of which it is a natural component, and from enotechnic tasks during the fermentation process; in warm areas, the use of this acid is allowed to adjust the pH of the musts before fermentation. Its concentration decreased throughout the ageing process in all the wines studied due to the precipitation of potassium bitartrate and calcium tartrate. The tartaric acid comes from the grapes and from the addition of tartaric acid during the harvest in order to adjust the pH of the musts in warm areas. Its concentration decreased throughout the ageing process in all the wines studied because of the precipitation of calcium tartrate and potassium bitartrate [7,44].

### 3.3. Acetaldehyde, Diethyl-Acetal, Methanol and Higher Alcohols of Oloroso and Palo Cortado Sherry Wines

The initial aldehyde content (acetaldehyde and its diethyl acetal), which is mainly a consequence of the fermentation process, is slightly higher in the Palo Cortado Sherry wine (79.3 mg/L) than in the Oloroso Sherry wine (70.7 mg/L), although no major differences are observed. This content evolves increasingly with ageing (Table 6) due to the effect of alcohol oxidation in the *Criaderas y Solera* systems.

As for methanol, an increase in methanol is observed during the ageing of Oloroso and Palo Cortado Sherry wines. This compound is produced in the wines during must fermentation and is influenced by the pressure used for the pressing of the grapes (a higher pressure favors a greater presence of methanol). The increment of methanol in the dry wines, Oloroso and Palo Cortado Sherry wines, is mainly due to the *merma*, as well as to transfers from the wood (Table 6) [17].

A similar evolution is observed in the higher alcohols studied (n-propanol, isobutanol, 2-methyl-1-butanol and 3-methyl-1-butanol): they all evolve increasingly during the ageing of the two types of sherry wines studied, mainly due to an increasing concentration of these compounds as an effect of the *merma*. These higher alcohols originate during the alcoholic fermentation through different metabolic pathways from the decarboxylation/deamination of amino acids [44,46], as well as through the small contribution from the 95.4% ABV wine alcohol used for the fortification of Oloroso and Palo Cortado Sherry wines.

Hexanol content was low in all the wines studied, with values between 0.80 and 1.17 mg/L in both Oloroso and Palo Cortado Sherry wines, and remained stable through the oxidative ageing. 2-phenylethanol is a compound that originates from phenylalanine during the alcoholic fermentation by yeasts [48] and is concentrated by the effect of the *merma* that takes place during oxidative ageing.

For most of the compounds that were examined, significant differences were observed between the different ageing scales of either of the wines studied, except for those scales where no notable evolution during ageing, such as hexanol, could be observed.

### 3.4. Major Esters Present in Oloroso and Palo Cortado Sherry Wines

Ethyl acetate stands out among all the esters studied (Table 7), since it is one of the compounds that evolved the most in the wines studied, and in an increasing manner with ageing. On the one hand, the ethanol in the medium undergoes an oxidative process that produces acetic acid [33], and this acetic acid, in turn, is released from the cask wood [17]. The esterification of acetic acid with the ethanol in the medium gives rise to ethyl acetate, which is also concentrated by the *merma* effect (Table 2). The ethyl acetate values in the Solera de Palo Cortado and Oloroso Sherry wines are very similar (325.3–323.7 mg/L).

Esters are produced by yeasts during the alcoholic fermentation of the must as by-products of sugar metabolism and represent one of the most important groups of fermentative aroma compounds that carry the organoleptic characteristics of wine [49]. Furthermore, some esters may increase their concentration by the chemical esterification of organic and/or fatty free acids with ethanol or with other alcohols that are present in the wine during its long-term oxidative ageing [7].

The highest concentrations of ethyl lactate and diethyl succinate are found in the Palo Cortado Sherry wine, reaching values of 370.17 and 171.63 mg/L, while the values found in the Solera de Oloroso Sherry wine are 203.65 and 89.67 mg/L, respectively.

In the two types of wine studied, fatty acid ethyl esters (ethyl hexanoate, ethyl octanoate, ethyl decanoate, ethyl dodecanoate, ethyl tetradecanoate and ethyl hexadecanoate) show a rather flat evolution with oxidative ageing, except for ethyl octanoate, which increases slightly with age. For this reason, most of them do not show significant differences between the different ageing scales of the same sherry wine. The contents of fatty acid ethyl esters exhibit a similar behavior to that found by our research group in other types of sherry wines, such as Fino or Amontillado [7]. Palo Cortado Sherry wine has a greater content of fatty acid ethyl esters than Oloroso Sherry wine, due to its initial biological ageing stage [4,50,51].

The effect of the *merma* cannot be appreciated with regard to the concentrations of fatty acid ethyl esters in the old wines, since these esters are highly insoluble below 45% ABV.

### 3.5. Phenolic Composition and Folin–Ciocalteau Index of Oloroso and Palo Cortado Sherry Wines

The FCI increased steadily with the ageing of the two types of wine studied (Table 8) as a result of the transfer of compounds from the casks wood, the effect of the *merma* and the diverse chemical reactions that take place during the ageing process involving the polyphenolic compounds from the grapes (Table 8) [17,52].

The starting FCI values in young Palomino fortified wine for Palo Cortado (246 mg/L gallic acid equivalent) are lower than for Oloroso (290 mg/L gallic acid equivalent). This is due to the fact that in the case of the Palo Cortado, the young wine was initially selected to age as Fino Sherry wine (a very pale, delicate type of sherry wine and where the least oxidative capacity of the wine is desired, therefore, with low polyphenol content), whose first stage of ageing is Sobretabla, precisely where winemakers find the future wines selected for Palo Cortado; while, for the Oloroso, those young wines with more structure and therefore with a higher FCI (higher polyphenolic content) are chosen.

Total polyphenol contents are higher in the Oloroso Sherry wine than in the Palo Cortado Sherry wines. The Oloroso Sherry wine reached 791 milligrams of gallic acid per liter compared to 598 milligrams of gallic acid per liter in the Palo Cortado Sherry wine. Significant differences regarding this parameter were observed between all the ageing scales of the two sherry wines studied.

With respect to grape-specific phenolic compounds, it has been observed that tartaric acid derivatives decreased with ageing time, possibly due to hydrolytic processes, since their free polyphenolic structure increases at the time of hydrolysis (caffeic acid, p-coumaric acid and ferulic acid), although later, in long aged wines, a reduction in caffeic acid and ferulic acid content can also be observed that does not seem to affect p-coumaric acid.

During the oxidative ageing of the two types of wines studied, there is a significant rise in the contents of 5-hydroxymethylfurfural and furfural, which is attributable to Maillard reactions (which are significantly influenced by the large number of ageing years of these wines), as well as that yielded by the wood itself (furfural comes from the dehydration and cyclization of the pentoses during the heating of the boot during its manufacture, while 5-hydroxymethylfurfural has its origin in the thermal degradation of hexoses and rhamnose) [17,53]). In all the wines, the *merma* phenomenon (Table 2) also contributed to the increasing concentrations of these two furfurals.

### 3.6. Evolution of Color during the Ageing of Oloroso and Palo Cortado Sherry Wines

The absorbance at 470 nm can compare the yellow–old gold–topaz–amber–mahogany color range. This parameter increases with time in the two *Criaderas y Solera* systems studied (Table 9) due to the effect of the oxidative processes and/or polymerization of certain compounds in the wine, generally with polyphenolic structure, quinones, etc., which are initially colorless and become tinted [54,55]. The presence of ions such as iron and copper in the wines has an influence on these oxidative processes, as they act as catalysts: the iron content in the wines studied is between 1 and 5 mg/L for both Oloroso and Palo Cortado Sherry wines, while copper reaches just between 0.10 and 0.75 mg/L. The lowest values correspond to the younger wines and the highest to the oldest Criaderas and the Soleras. In addition, there is a transfer of compounds from the wood of the casks with ageing time, which contributes to intensifying their color. The absorbance values registered presented significant differences between the ageing scales of all the wines studied.

### 3.7. Principal Component Analysis of Oloroso and Palo Cortado Sherry Wines

A principal component analysis was performed using the 55 physicochemical variables studied. The PCA was carried out with the standardized variables and following the criterion of a minimum eigenvalue of 1.0 for the extraction of the factors. The results of the analysis showed that five factors explained 91.41% of the total variability of the data.

**Figure 2:**
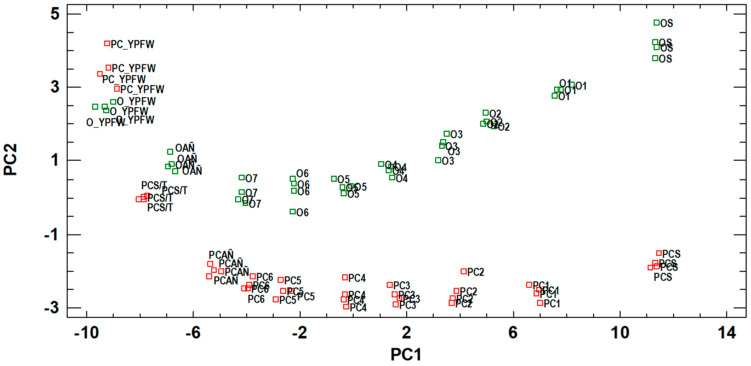
Visual representation of the wines on the two-dimensional plane defined by PC1 (71.03%) and PC2 (9.18%). PC: Palo Cortado; O: Oloroso; YPFW: Young Palomino Fortified Wine; AÑ: *Añadas*; S/T: *Sobretabla*; S: Solera; the numbers next to the wine names indicate the Criadera scale.

The projection of the samples on the plane of principal components (PC) 1 and 2 allows for observation of two clear groupings (Figure 2) according to the type of Sherry wine.

Along PC1 (*X*-axis), the samples are ordered from left to right according to the age of the samples as follows: For the Oloroso Sherry wine, the rank was Young Palomino Fortified Wine > *Añadas* > 7th Cra. > 6th Cra. > 5th Cra. > 4th Cra. > 3rd Cra. > 2nd Cra. > 1st Cra. > Solera, and for the Palo Cortado Sherry wine, the order was Young Palomino Fortified Wine > *Sobretabla* > *Añadas* > 6th Cra. > 5th Cra. > 4th Cra. > 3rd Cra. > 2nd Cra. > 1st Cra. > Solera. Therefore, PC1 is related to ageing. On the Y axis, PC2, the ranking of the samples according to the type of sherry wine can be observed: the Oloroso Sherry wines are located in the positive area of this component, while the Palo Cortado Sherry wines are located in the negative area of this component (except for the initial one).

Considering this, although both wines are aged through oxidative ageing, the Palo Cortado starts its ageing through a biological process, and this factor can differentiate Palo Cortado from Oloroso wine. These ranks and influences are supported by consulted bibliography, since they had already been observed by our research group in previous works [5,7].

Table 10 shows the weight of components 1 and 2, and only those with r > 4 (the most significant) have been included. PC1 shows positive and high correlations with many of the variables studied, among which are those compounds whose concentrations increase significantly during ageing, either due to the effect of the *merma* (Table 2), such as most of the higher alcohols, or due to wood yields, such as acetic acid (with its subsequent esterification into ethyl acetate) or other phenolic compounds. Those with a high and negative correlation, such as caffeic acid or derivatives, are the acids from the grapes, with a polyphenolic and tartaric structure that, during ageing, hydrolyze and disappear.

PC2 is related to biological ageing and shows positive and high correlations with malic acid and pH. Malic acid is metabolized through malolactic fermentation by lactic acid bacteria, which cohabits with the veil of flor during the early stages of biological ageing. Therefore, its content is greater in Oloroso Sherry wines (positive correlation) than in Palo Cortado Sherry wines, which undergoes a biological ageing stage at the beginning of the process. The reduction of the pH is independent from biological ageing, as this is also observed in oxidative ageing. In our case, a drop in the pH of the young fortified wine compared to the *Sobretabla* wine can be observed, since the medium was set at 15% ABV, where the potassium bitartrate and calcium tartrate precipitated and caused a drop in the pH to reach its chemical equilibrium (PC2 turned into negative values). Then, as the Palo Cortado is fortified again at 17% ABV, a more negative PC2 value could be observed in the *Añadas*, which could be explained by the new precipitations and the pH drop after reaching the new chemical equilibrium. This explains the fact that there is only one step distance from the young fortified wine to the Oloroso *Añadas*, while for Palo Cortado, the figure shows two steps: the first fortification at 15% ABV (biological ageing) and the second at 17% ABV (oxidative ageing). High negative correlations have also been observed with acetaldehyde, ethyl octanoate and citric acid. Yeasts produce acetaldehyde, and therefore, Palo Cortado Sherry wines have a higher content of this compound (negative area of the component). When the wines selected for the production of Palo Cortado are removed from the *Sobretabla* casks and fortified at 17% ABV for the *Añadas* ageing process, the ethyl octanoate content increases, which is probably due to the cell lysis of the dragged yeasts, which release this compound into the medium [7]. The citric acid is consumed by the yeasts of the veil of flor; therefore, it should be higher in the Oloroso and was expected to have a positive influence. However, it presents a negative influence, which may be explained by the narrow difference between the citric acid content of the young fortified wine and that of the *Sobretabla* wine. Therefore, the variables that are closely related to some biological activity are those with the greatest weight in PC2 extracted from the model (Figure 2).

The older the wine, the more evident differentiation between the two groups of sherry wines. This was confirmed sensorially during the tasting sessions that were carried out with the Oloroso and Palo Cortado wines of 12 and more than 30 years of age (Section 3.9).

Principal components 3, 4 and 5 are not so relevant, since principal components 1 and 2 explain more than 80% of the total variability of the data explained by the model. The analysis of the factor loading matrix confirms the reading of the projection of the samples on the plane of principal components 1 and 2 (Figure 2), which differentiates between the different wines according to their ages and to the ageing system employed.

### 3.8. Multiple Linear Regression Analysis to Predict the Age of the Sherry Wines

A multiple linear regression (MLR) approach was used to determine which of the variables studied had a direct correlation with the age of the wine and to develop models that could be used to predict or confirm the age of a particular sherry wine.

The strategy followed for this purpose consisted of finding a model that used the fewest possible number of independent variables, with a maximum of 5.

With this objective, the initial study was carried out grouping the variables according to families of compounds (glycerol and organic acids, phenolic compounds, volatile compounds (alcohols and esters) and the oenological parameters studied (dry matter, reducing matter, sulfate, calcium, potassium and absorbance at 470 nm).

The “Multiple Regression Models” procedure of StatGraphics 19 was applied, with the aim of finding the best model for one of the groups of variables.

This procedure combines all predictor variables from 0 to 5 and computes the statistics for evaluation, namely the degrees-of-freedom-adjusted R2 values, mean square error (MSE), *p* values of the predictor variables, the model for 95% significance value, Mallows’ Cp, and the Schwarz–Bayesian Information Criterion (SBIC).

The criteria for selecting the best model for all cases are the following: values less than 0.05 in the *p* values of the predictor variables and of the model, values close to 100 in R2, and values as small as possible in the Cp of Mallows and SBIC.

Once the best model for each group of variables was determined, in a second stage, and following the same strategy, the possibility of combining the different groups of variables was verified. At this stage, only those that were previously selected as predictor variables in the previous models were considered as predictor variables. The two systems of *Criaderas y Solera* were studied separately in order to adapt the models to the two types of sherry wines under study. From each of the corresponding data matrices, 8–9 cases (depending on the data matrix corresponding to each sherry wine) were drawn and used to validate the model after it had been built.

#### 3.8.1. Determining the Average Age of the Ageing Oloroso Sherry Wines

The best models obtained for the Oloroso Sherry wine samples were included in Table 11. As can be seen, the O5 model is the best of the five obtained. This model presents a significance level greater than 95% and an R2 (adjusted by GL) that explains 99.9247% of the variance.

The five variables that are part of the equation of the O5 model (citric acid, succinic acid, lactic acid, ferulic acid and volatile acidity) are closely linked to the oxidative ageing of this wine.

Citric, succinic and lactic acids have an upward trend during oxidative ageing (Table 5) due to the effect of the *merma*. In addition, significant differences between the levels of these three acids can be observed for each ageing scale of the Oloroso Sherry wines, which are differentiating indicators of its ageing stage.

Ferulic acid, which is one of the specific polyphenols in grapes, increasingly evolves during the first stages of the ageing process (Table 8), reaching its maximum concentration in the 6th Criadera of Oloroso Sherry wine (0.94 mg/L). This is due to the concentrations of ferulic acid as a result of the *merma* and to the hydrolysis of fertaric acid. Slight decreases in the concentration of fertaric acid are observed with ageing time, possibly due to hydrolytic processes. This explains the negative sign in the equation. In any case, the evolution of ferulic acid in the last ageing scales is not clear, since its concentrations due to the *merma* and the contribution that may occur through the oxidation of the coniferaldehyde yielded by the wood [16] are compensated by the hydrolysis reactions that may take place.

The fifth variable in the equation is the volatile acidity of the wines, which is a clear marker of their age. This parameter increases (Table 4) because of the oxidation of the alcohol in the medium and because of the acetic acid transferred from the wood, as well as by the effect of the *merma*, so that the longer the ageing time, the higher the volatile acidity of the wine.

In order to validate this model, the cases that had been previously extracted from the set of samples were examined. The results, which are shown in Table 12, result in a high-precision model where the greatest error corresponds to the oldest wines.

#### 3.8.2. Determining the Average Age of the Ageing Palo Cortado Wines

The best models obtained for the Palo Cortado Sherry wine samples are shown in Table 13. As can be seen, the PC5 model is the best of the five obtained. This model presents a significance level above 95% and an R2 (adjusted for GL) that explains 99.9142% of the variance. The five variables that are part of the equation of the PC5 model (lactic acid, total acidity, volatile acidity, p-coumaric acid and 5-Hydroxymethylfurfural) are related to the oxidative ageing of Palo Cortado Sherry wines and, some of them, to the first stage of the biological ageing of this wine.

Lactic acid (Table 5) owes its presence to the metabolic activity of the yeasts during the alcoholic fermentation stage [46]. In the case of Palo Cortado Sherry wine, during its biological ageing stage (*Sobretabla*), a significant increase in the lactic acid from malolactic fermentation is observed, which during oxidative ageing, is also increased as an effect of the *merma*. This is why lactic acid is the predominant acid in the oldest Palo Cortado wines.

Total acidity (Table 4) increases with ageing due to the oxidation of the ethanol in the medium, which results in an increasing acetic acid content [32], the concentration of organic acids due to the *merma* (Table 2) and the acidic substances contributed by the wood [17].

As occurred in the model used to predict the age of Oloroso Sherry wines, volatile acidity appears in this model, which similarly to total acidity, increases during the ageing process (Table 4) due to the alcohol oxidation processes in the medium, to the acetic acid transferred from the wood and to the effect of the *merma*.

P-coumaric acid (Table 8) increases during ageing. This is explained by the hydrolytic reactions that involve tartaric derivatives, such as cis-p-coutaric acid and trans-p-coutaric acid, which increase the wines’ free polyphenolic structure (p-coumaric acid). The effect of the *merma* also adds to this increment.

5-hydroxymethylfurfural (Table 8) increased markedly during ageing as a consequence of the Maillard’s reactions that take place in the medium, the wood yields and the effect of the *merma*.

In order to validate this model, the cases that had been previously extracted from the pooled sample were examined again, and the results are shown in Table 14.

### 3.9. Tasting Sessions of Oloroso and Palo Cortado Sherry Wines

Table 15 presents the mean and standard deviations of the scores granted to the five wines for each sensory descriptor by the panel. The standard deviations lower than 1 in all the cases suggested the high homogeneity of the judging panel. To confirm this, the data were subjected to an analysis of variance where each judge was taken as the factor. The *p* values obtained (ptaster) were all well above 0.05, which clearly indicates the non-significance of this factor, i.e., all the judges made very similar judgments, with no significant differences between them.

**Figure 3:**
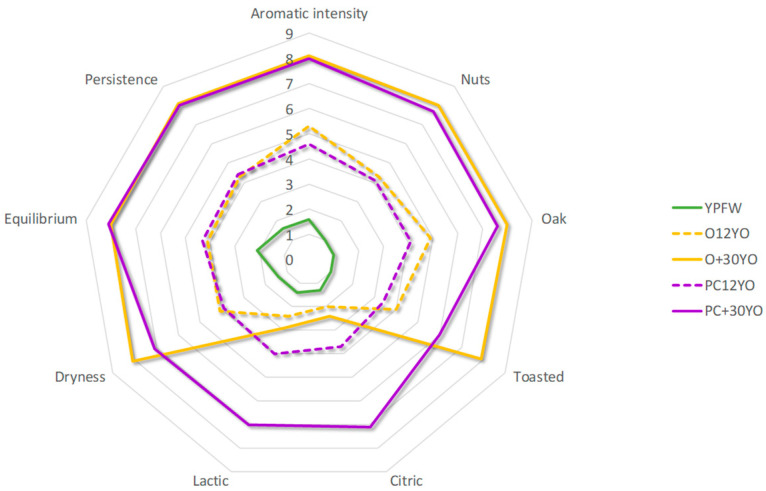
Spider-web graph showing the average sensory scores granted by the tasting panel to the evaluated wines.

When the analysis of variance was applied to the data but, this time, using the sample as the variance factor, it was observed that all the descriptors, either olfactory or olfactory–gustatory, exhibited some differentiating capacity (*p* value < 0.05). Regarding the young fortified Palomino wine, the sensory scores for all the descriptors were low (in the 1–2 range), even though the ageing time translated into significant scoring increments for nearly all of them, regardless of the wines’ evolution into either Oloroso or Palo Cortado. Thus, the aged Oloroso and Palo Cortado wines were perceived as having a greater aromatic intensity, with clearer hints of nuts, oak and toast, and a drier, more persistent and balanced mouth feel. With regard to these descriptors, only the toasted aroma and the dryness in the mouth were perceived differently with time, so that they were significantly higher for the very old Oloroso wine (O+30YO), reaching around eight points. The citrus and lactic notes, however, showed a different behavior as follows: they started at the low values of the young fortified wine, while the differences in intensity of these notes in the Oloroso wines were exclusively related to whether they had entered or not the ageing system, but the ageing time did not seem to have any effect (scores hovered around low–medium values within the 2–3 range). Contrarily, a direct relation with ageing was confirmed for the Palo Cortado wines, so that maximum intensities, hovering around seven points for citrus or milky notes, were perceived in the more than 30 years old wine. The spider-web graph (Figure 3) visually compares the sensory profiles of the different wines evaluated.

**Figure 4:**
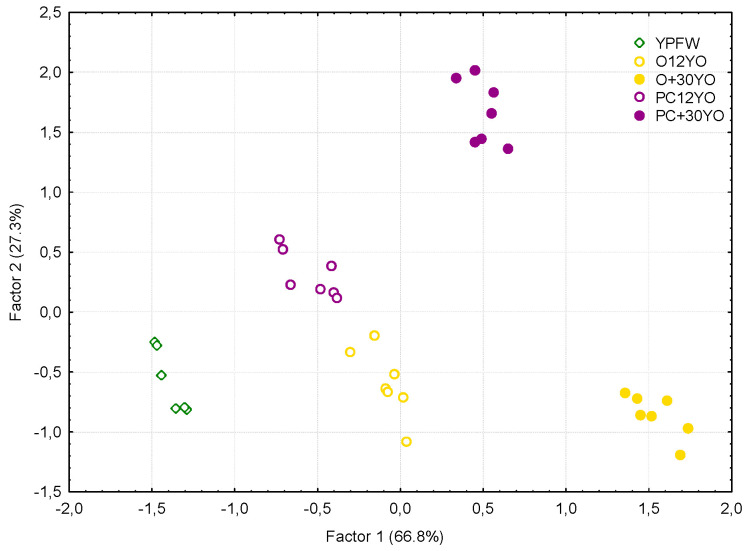
Projection of the wines according to the factorial analysis of the data on the plane formed by Factors 1 and 2.

The multivariate processing of the data by factorial analysis led to similar results. The homogeneity and differentiating capacity of the judges was evidenced by the clustering of the scores for each sample and by the score distances between such clusters. The factors were extracted according to the means of the principal components. Those with an eigenvalue greater than 1 were selected, and the interpretation or identification of the variables behind each one was facilitated after the varimax rotation of the factors. The analyses determined two factors that explained 94.1% of the total variability of the data. According to the projection of the wines on the new space (Figure 4), Factor 1, which explained 66.8% of the variability, was related to ageing time in cask, given that the wines were ranked from left to right starting with the young fortified wine, moving through the 12-year-old wines and finally reaching the oldest 30-year-old wines.

**Figure 5:**
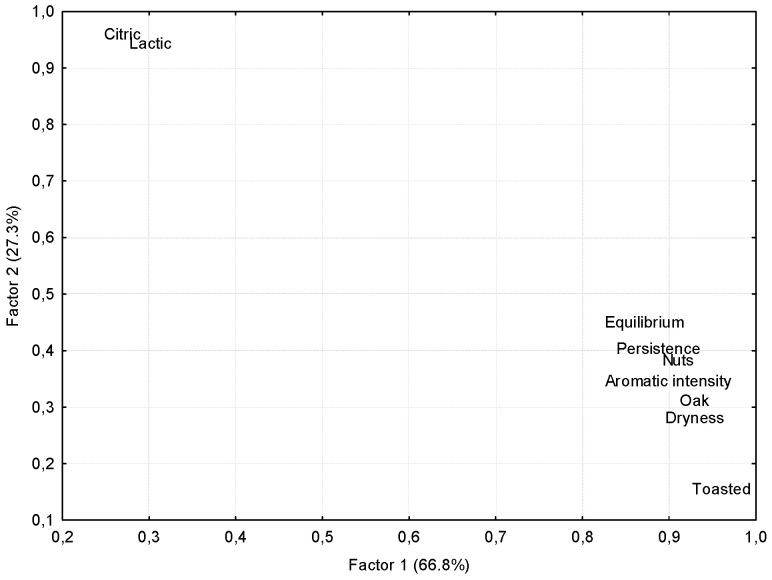
Weight of the sensory descriptors with respect to the first factors in the factorial space obtained.

Figure 5 shows the weights of the sensory descriptors in the factorial space obtained from the first two factors, which should allow us to confirm the relevance of these factors when attempting to establishing a differentiating profile for the various wines.

Thus, we can observe the high correlation of Factor 1 with the olfactory note of oak, as expected, but also with the aromatic intensity, the olfactory notes of nuts and toasted fruits, and the dryness, persistence and balance in the mouth. All of these notes presented correlation coefficients greater than 0.85 with Factor 1. These are the descriptors that increased during the ageing of both wines. Some of them presented a slightly greater increment in the Oloroso wines, which is reflected by their right-shifting positions with respect to those corresponding to the Palo Cortado wines of the same age. As can be seen in Figure 5, Factor 2, which explains 27.3% of the total variability of the data, presented a high positive correlation with lactic and citrus olfactory notes (r > 0.9), which from a univariate point of view, clearly differentiated the Oloroso from the Palo Cortado wines, as above explained. It can be observed from the projection of the samples on the F1-F2 plane (Figure 4), that after starting from the same young fortified Palomino wine (YPFW), the Palo Cortado wines reach higher F2 values with respect to those corresponding to the Oloroso wines, with the distance between them increasing with ageing time. It seems clear, therefore, that Factor 2 is related to the winemaking procedures, but also to its evolution in the ageing system, so that only the Palo Cortado wines are perceived as more citric and lactic as they grow older.

## 4. Conclusions

Oloroso and Palo Cortado are two sherry wines that age under oxidative ageing and are differentiated by the peculiarities acquired during the first ageing stage of Palo Cortado under biological ageing. The reminiscences from this first ageing stage under a veil of flor are revealed by the presence of certain compounds involved in the metabolism of the yeasts as well as in that of the bacteria that cohabit with yeasts and that have an impact on the chemical composition of this wine. Under an oxidative environment, the contributions from the wood and the growing concentration of the different compounds due both to the effect of the *merma* and to the oxidative reactions gain an increasing significance. All of this has a substantial impact on the organoleptic characteristics of these sherry wines, as could be confirmed during the tasting sessions that have been conducted. The sensorial analysis revealed that the lactic and citrus notes were the most relevant when it comes to differentiating between Oloroso and Palo Cortado, with greater differences being found as the wines aged. These notes are closely related to the singular first stage of biological ageing that Palo Cortado is subjected to. In the case of Oloroso Sherry wines, those of older age present a significant predominance of toasted aroma and dryness in the mouth.

These trends have been appreciated in the principal component analyses that have been carried out, where a number of clusters according to biological ageing (Palo Cortado), oxidative ageing (Oloroso) and their different ageing times could be observed. The multiple linear regression analyses revealed a strong correlation between the age of the sherry wines and the determined parameters, so that two different models were generated: one for the Oloroso Sherry wines and the other one for the Palo Cortado Sherry wines. The models could estimate the age of either type of wine at over 99% confidence. This represents a considerable development regarding the monitoring or quality control procedures in the industry, since only five parameters are required by these models to determine the age of these wines.

## Figures and Tables

**Table 1:** Environmental conditions of the cellar where the experiments were carried out.

Month	Average Humidity (%)	Average Temperature (°C)
January	79.0 ± 3.9	12.5 ± 3.0
February	77.5 ± 5.3	12.0 ± 1.8
March	79.5 ± 3.7	14.3 ± 1.5
April	71.3 ± 3.8	14.5 ± 3.5
May	69.0 ± 3.5	18.0 ± 1.8
June	68.5 ± 3.1	21.5 ± 1.3
July	65.8 ± 3.5	24.0 ± 3.9
August	66.8 ± 2.9	24.8 ± 4.1
September	75.5 ± 4.8	24.3 ± 2.5
October	76.5 ± 4.5	22.5 ± 2.6
November	75.8 ± 5.3	18.8 ± 3.3
December	78.3 ± 5.6	15.3 ± 2.2

Mean values ± standard deviation (*n* = 4) are shown.

**Table 2:** Effect of a *merma* of 3% in volume with ageing for the different average ageing times studied. Data extracted from reference [7].

Average Age (Years)	Volume of Wine in the Casks (%)	Concentration Increase in Non-Volatile Compounds (%)
1	97.00	103
2	94.09	106
3	91.27	110
4	88.53	113
5	85.87	116
8	78.37	128
12	69.38	144
16	61.43	163
20	54.38	184
30	40.10	249
40	29.57	338
50	21.81	459
60	16.08	622

**Table 3:** Odor and flavor patterns and descriptors used during the tasting sessions.

Descriptor	Definition	Pattern (Oloroso and Palo Cortado Sherries)
Odor (nose)		
Aromatic intensity	Intensity of all the positive aromatic notes of the wine	Oloroso Sherry and Palo Cortado Sherry wines with 50 and 60 years old of average ageing (special casks)
Nuts	Nut aromas, mainly walnut and other nuts in Oloroso Sherry wine and hazelnut and other nuts in Palo Cortado Sherry wine	Oloroso Sherry and Palo Cortado Sherry wines with 50 and 60 years old of average ageing (special casks)
Oak	Characteristic aromas of oak, with hints of vanilla and spices	Oloroso Sherry wine with 50 years old of average ageing (special casks)
Toasted	Characteristic aromas of toffee, honey, syrup, roasted, coffee, bitter cocoa, cocoa, etc.	Oloroso Sherry wine with 50 years old of average ageing (special casks)
Citric	Bitter orange and dried citrus peel aromas.	Palo Cortado Sherry wine 60 years old of average ageing (special casks)
Lactic	Aroma reminiscent of dairy, butter, etc.	Palo Cortado Sherry wine 60 years old of average ageing (special casks)
Flavor (mouth)		
Dryness	Dry sensation of the wine, without astringency on the palate	Oloroso Sherry wine with 50 years old of average ageing (special casks)
Equilibrium	Overall positive evaluation of the sensations in the mouth, with a good integration of the alcohol and acidity, without astringency or harshness, but with the aromatic reminder of the oak, as appropriate for an oak-aged wine	Oloroso Sherry and Palo Cortado Sherry wines with 50 and 60 years old of average ageing (special casks)
Persistence	Time evaluation of the positive olfactory–gustatory notes remaining after the final sip	Oloroso Sherry and Palo Cortado Sherry wines with 50 and 60 years old of average ageing (special casks)

**Table 4:** Parameters of Oenological Control: (a) alcoholic strength (% ABV), density (g/L), pH, total acidity (g TH_2_/L), volatile acidity (g AcH/L), glycerol (g/L), in the Oloroso and Palo Cortado Sherry wines studied. (b) total dry extract (g/L), total sulfur dioxide (mg/L), sulphates (g K_2_SO_4_/L), calcium (mg/L), potassium (mg/L), reducing substances (g/L) and sugar-free extract (g/L) in the Oloroso and Palo Cortado Sherry wines studied.

(a)	Alcoholic Strength	Density	pH		Total Acidity	Volatile Acidity	Glycerol
Oloroso Sherry Wine							
Y.P.F.W.	17.63 ± 0.21 ^a^	984.47 ± 0.31 ^a^	3.30 ± 0.06 ^a^		5.40 ± 0.18 ^a^	0.30 ± 0.06 ^a^	6.15 ± 0.29 ^a^
*Añada*	18.13 ± 0.15 ^b^	984.47 ± 0.12 ^a^	3.32 ± 0.03 ^a^		4.80 ± 0.05 ^b^	0.39 ± 0.03 ^b^	6.48 ± 0.06 ^b^
7th Cra.	18.47 ± 0.06 ^c^	984.77 ± 0.12 ^b^	3.37 ± 0.01 ^b^		5.05 ± 0.05 ^c^	0.45 ± 0.02 ^c^	6.89 ± 0.04 ^c^
6th Cra.	18.93 ± 0.06 ^d^	984.90 ± 0.10 ^b,c^	3.37 ± 0.02 ^b^		5.41 ± 0.03 ^a^	0.55 ± 0.02 ^d^	7.52 ± 0.04 ^d^
5th Cra.	19.37 ± 0.06 ^e^	985.03 ± 0.15 ^c^	3.37 ± 0.02 ^b,c^		5.62 ± 0.03 ^d^	0.62 ± 0.01 ^e^	8.00 ± 0.05 ^e^
4th Cra.	19.77 ± 0.06 ^f^	985.53 ± 0.21 ^d^	3.40 ± 0.02 ^c,d,e^		6.28 ± 0.08 ^e^	0.80 ± 0.02 ^f^	8.75 ± 0.10 ^f^
3rd Cra.	20.07 ± 0.06 ^g^	986.00 ± 0.20 ^e^	3.40 ± 0.02 ^b,c,d^		7.04 ± 0.17 ^f^	0.90 ± 0.03 ^g^	9.90 ± 0.17 ^g^
2nd Cra.	20.60 ± 0.10 ^h^	986.67 ± 0.15 ^f^	3.41 ± 0.01 ^d,e,f^		7.32 ± 0.05 ^g^	1.03 ± 0.03 ^h^	10.73 ± 0.08 ^h^
1st Cra.	21.77 ± 0.15 ^i^	986.60 ± 0.17 ^f^	3.43 ± 0.02 ^e,f^		8.02 ± 0.13 ^h^	1.23 ± 0.04 ^i^	12.91 ± 0.08 ^i^
*Solera*	23.73 ± 0.15 ^j^	986.37 ± 0.06 ^g^	3.44 ± 0.02 ^f^		9.81 ± 0.11 ^i^	1.37 ± 0.06 ^j^	16.22 ± 0.08 ^j^
Palo Cortado Sherry Wine							
Y.P.F.W.	15.63 ± 0.15 ^a^	986.10 ± 0.30 ^a^	3.20 ± 0.05 ^a^		5.99 ± 0.23 ^a^	0.25 ± 0.08 ^a^	7.15 ± 0.71 ^a^
S/T W. *	14.93 ± 0.20 ^b^	986.23 ± 0.21 ^a^	3.08 ± 0.04 ^d^		5.26 ± 0.19 ^b^	0.35 ± 0.03 ^b^	4.81 ± 0.14 ^b^
*Añada*	17.13 ± 0.10 ^c^	985.23 ± 0.12 ^e^	3.05 ± 0.01 ^e^		5.16 ± 0.07 ^b^	0.35 ± 0.03 ^b^	5.66 ± 0.02 ^c^
6th Cra.	17.40 ± 0.10 ^d^	985.50 ± 0.17 ^c^	3.05 ± 0.02 ^d,e^		5.32 ± 0.08 ^b^	0.36 ± 0.02 ^b^	7.16 ± 0.10 ^a^
5th Cra.	17.75 ± 0.07 ^e^	985.70 ± 0.10 ^c,d^	3.07 ± 0.02 ^d,e^		5.53 ± 0.06 ^c^	0.38 ± 0.02 ^b^	7.87 ± 0.06 ^d^
4th Cra.	18.43 ± 0.12 ^f^	986.07 ± 0.06 ^a,d^	3.09 ± 0.01 ^c,d^		6.26 ± 0.05 ^d^	0.45 ± 0.02 ^c^	9.46 ± 0.06 ^e^
3rd Cra.	19.25 ± 0.13 ^g^	985.87 ± 0.06 ^d^	3.11 ± 0.02 ^b,c^		6.69 ± 0.07 ^e^	0.53 ± 0.02 ^d^	10.27 ± 0.10 ^f^
2nd Cra.	19.87 ± 0.12 ^h^	985.77 ± 0.06 ^c^	3.13 ± 0.01 ^b^		6.93 ± 0.10 ^f^	0.71 ± 0.03 ^e^	11.71 ± 0.15 ^g^
1st Cra.	22.02 ± 0.19 ^i^	984.70 ± 0.26 ^b^	3.19 ± 0.01 ^a^		7.74 ± 0.13 ^g^	0.91 ± 0.02 ^f^	13.49 ± 0.09 ^h^
*Solera*	23.53 ± 0.15 ^j^	984.80 ± 0.26 ^b^	3.24 ± 0.03 ^f^		9.34 ± 0.24 ^h^	1.26 ± 0.04 ^g^	16.81 ± 0.23 ^i^
**(b)**	**Total Dry** **Extract**	**Total** **SO_2_**	**Sulfates**	**Calcium**	**Potassium**	**Reducing** **Substances**	**Sugar-Free** **Extract**
Oloroso Sherry Wine							
Y.P.F.W.	21.63 ± 0.25 ^a^	77 ± 8 ^a^	0.90 ± 0.13 ^a^	94 ± 17 ^a,b^	1265 ± 72 ^a^	1.77 ± 0.21 ^a^	19.87 ± 0.42 ^a^
*Añada*	22.93 ± 0.25 ^b^	53 ± 2 ^b^	0.87 ± 0.04 ^a^	93 ± 2 ^a^	889 ± 15 ^b^	2.50 ± 0.30 ^b^	20.43 ± 0.49 ^a^
7th Cra.	24.57 ± 0.40 ^c^	40 ± 4 ^c^	0.93 ± 0.03 ^a^	92 ± 1 ^a^	870 ± 8 ^b^	3.17 ± 0.25 ^c^	21.40 ± 0.66 ^b^
6th Cra.	26.40 ± 0.17 ^d^	28 ± 1 ^d^	1.07 ± 0.04 ^b^	96 ± 2 ^a,b^	979 ± 6 ^c^	3.53 ± 0.21 ^c^	22.87 ± 0.15 ^c^
5th Cra.	27.90 ± 0.50 ^e^	17 ± 4 ^e^	1.23 ± 0.02 ^c^	101 ± 3 ^b^	1111 ± 19 ^d^	4.33 ± 0.31 ^d^	23.57 ± 0.21 ^d^
4th Cra.	30.40 ± 0.53 ^f^	7 ± 1 ^f^	1.36 ± 0.03 ^d^	113 ± 4 ^c^	1310 ± 16 ^e^	6.10 ± 0.30 ^e^	24.30 ± 0.26 ^e^
3rd Cra.	32.47 ± 0.57 ^g^	4 ± 1 ^f,g^	1.45 ± 0.03 ^e^	121 ± 4 ^d^	1410 ± 19 ^f^	7.23 ± 0.45 ^f^	25.23 ± 0.38 ^f^
2nd Cra.	35.60 ± 0.53 ^h^	1 ± 1 ^g,h^	1.66 ± 0.03 ^f^	127 ± 3 ^d^	1686 ± 12 ^g^	8.83 ± 0.55 ^g^	26.77 ± 0.35 ^g^
1st Cra.	39.03 ± 0.68 ^i^	1 ± 1 ^g,h^	1.94 ± 0.04 ^g^	136 ± 5 ^e^	2228 ± 31 ^h^	10.23 ± 0.75 ^h^	28.80 ± 0.92 ^h^
*Solera*	43.67 ± 0.46 ^j^	0 ± 1 ^h^	2.38 ± 0.04 ^h^	139 ± 2 ^e^	2943 ± 43 ^i^	11.60 ± 0.30 ^i^	32.07 ± 0.74 ^i^
Palo Cortado Sherry Wine							
Y.P.F.W.	20.17 ± 0.40 ^a^	67 ± 11 ^a^	1.27 ± 0.23 ^a,d,e^	110 ± 21 ^a^	1136 ± 43 ^a^	2.63 ± 0.49 ^a^	17.53 ± 0.23 ^a^
S/T W. *	18.43 ± 1.02 ^b^	41 ± 3 ^b^	1.16 ± 0.08 ^b,c^	110 ± 12 ^a^	900 ± 39 ^b^	0.97 ± 0.21 ^b^	17.47 ± 0.81 ^a^
*Añada*	21.90 ± 0.00 ^c^	36 ± 2 ^c^	1.08 ± 0.03 ^c^	100 ± 4 ^b^	888 ± 12 ^b^	1.40 ± 0.36 ^c^	20.50 ± 0.36 ^b^
6th Cra.	23.73 ± 0.25 ^d^	26 ± 2 ^d^	1.18 ± 0.02 ^b,d^	96 ± 3 ^b^	847 ± 13 ^c^	1.93 ± 0.15 ^d^	21.80 ± 0.26 ^c^
5th Cra.	25.03 ± 0.40 ^e^	17 ± 2 ^e^	1.31 ± 0.03 ^e^	96 ± 2 ^b^	886 ± 10 ^b^	2.57 ± 0.15 ^a^	22.47 ± 0.40 ^d^
4th Cra.	27.87 ± 0.25 ^f^	9 ± 1 ^f^	1.54 ± 0.04 ^f^	98 ± 2 ^b^	978 ± 6 ^d^	3.67 ± 0.25 ^e^	24.20 ± 0.00 ^e^
3rd Cra.	29.70 ± 0.30 ^g^	5 ± 1 ^g^	1.63 ± 0.02 ^f,g^	103 ± 3 ^a,b^	1095 ± 30 ^e^	4.63 ± 0.12 ^f^	25.07 ± 0.40 ^f^
2nd Cra.	31.37 ± 0.40 ^h^	2 ± 1 ^g^	1.72 ± 0.03 ^g^	112 ± 3 ^a^	1241 ± 38 ^f^	5.83 ± 0.15 ^g^	25.53 ± 0.55 ^f^
1st Cra.	34.37 ± 0.25 ^i^	1 ± 1 ^g^	1.95 ± 0.03 ^h^	129 ± 3 ^c^	1695 ± 26 ^g^	6.80 ± 0.10 ^h^	27.57 ± 0.15 ^g^
*Solera*	39.17 ± 0.40 ^j^	0 ± 1 ^g^	2.41 ± 0.03 ^i^	149 ± 2 ^d^	2449 ± 44 ^h^	8.20 ± 0.20 ^i^	30.97 ± 0.21 ^h^

Mean values ± standard deviation (*n* = 4) are shown; ANOVA: for the same wine, different letters (a, b, c, d, e, f, g, h, i, j) indicate significant differences (*p* < 0.05). Y.P.F.W.: Young Palomino fortified wine; Cra: Criadera; S/T W. *: selected S/T wines for Palo Cortado; S/T: *Sobretabla*.

**Table 5:** Organic acids (mg/L) in the Oloroso and Palo Cortado Sherry wines studied.

	Citric Acid	Lactic Acid	Malic Acid	Succinic Acid	Tartaric Acid
Oloroso Sherry					
Y.P.F.W.	127 ± 44 ^a^	478 ± 92 ^a^	203 ± 62 ^a^	605 ± 24 ^a^	3757 ± 148 ^a^
*Añada*	145 ± 11 ^a,b^	671 ± 22 ^b^	199 ± 10 ^a^	670 ± 33 ^b^	2624 ± 80 ^b^
7th Cra.	156 ± 7 ^b,c^	823 ± 10 ^c^	209 ± 9 ^a,b^	710 ± 8 ^c^	2244 ± 56 ^c^
6th Cra.	168 ± 6 ^c^	880 ± 7 ^d^	228 ± 5 ^b,c^	795 ± 7 ^d^	1651 ± 107 ^d^
5th Cra.	187 ± 7 ^d^	978 ± 9 ^e^	250 ± 4 ^c^	835 ± 5 ^e^	1480 ± 43 ^e^
4th Cra.	202 ± 5 ^d^	1182 ± 15 ^f^	279 ± 6 ^d^	942 ± 8 ^f^	1347 ± 43 ^f^
3rd Cra.	229 ± 5 ^e^	1331 ± 18 ^g^	311 ± 4 ^e^	1076 ± 8 ^g^	1255 ± 23 ^g^
2nd Cra.	252 ± 5 ^f^	1658 ± 29 ^h^	339 ± 3 ^f^	1195 ± 7 ^h^	1163 ± 32 ^h^
1st Cra.	270 ± 6 ^g^	2018 ± 38 ^i^	374 ± 6 ^g^	1488 ± 9 ^i^	1045 ± 30 ^i^
*Solera*	283 ± 6 ^g^	2453 ± 45 ^j^	442 ± 11 ^h^	1860 ± 25 ^j^	949 ± 38 ^j^
Palo Cortado Sherry					
Y.P.F.W.	244 ± 67 ^a^	267 ± 71 ^a^	491 ± 116 ^a^	641 ± 82 ^a^	5121 ± 439 ^a^
S/T W. *	214 ± 32 ^a^	609 ± 51 ^b^	106 ± 24 ^b,c^	530 ± 35 ^b^	3325 ± 123 ^b^
*Añada*	278 ± 14 ^b^	804 ± 27 ^c^	101 ± 4 ^b^	609 ± 17 ^a^	2099 ± 22 ^c^
6th Cra.	323 ± 12 ^c^	1048 ± 9 ^d^	121 ± 2 ^b,c,d^	797 ± 14 ^c^	1611 ± 29 ^d^
5th Cra.	376 ± 7 ^d^	1214 ± 9 ^e^	133 ± 4 ^b,c,d,e^	846 ± 9 ^d^	1490 ± 10 ^d,e^
4th Cra.	428 ± 13 ^e^	1451 ± 8 ^f^	148 ± 2 ^c,d,e,f^	927 ± 7 ^e^	1361 ± 31 ^e,f^
3rd Cra.	468 ± 15 ^f^	1581 ± 7 ^g^	158 ± 4 ^d,e,f^	1078 ± 5 ^f^	1325 ± 19 ^e,f^
2nd Cra.	504 ± 9 ^g^	1777 ± 22 ^h^	170 ± 3 ^e,f^	1236 ± 8 ^g^	1282 ± 18 ^f^
1st Cra.	623 ± 10 ^h^	2231 ± 14 ^i^	191 ± 4 ^f,g^	1353 ± 19 ^h^	1215 ± 19 ^f,g^
*Solera*	758 ± 10 ^i^	2949 ± 22 ^j^	226 ± 14 ^h^	1761 ± 32 ^i^	1080 ± 11 ^g^

Mean values ± standard deviation (*n* = 4) are shown; ANOVA: for the same wine, different letters (a, b, c, d, e, f, g, h, i, j) indicate significant differences (*p* < 0.05). Y.P.F.W.: Young Palomino fortified wine; Cra: Criadera; S/T W. *: selected S/T wines for Palo Cortado; S/T: *Sobretabla*.

**Table 6:** (a) Acetaldehyde, acetaldehyde-diethylacetal, methanol and higher alcohols in mg/L in the Oloroso and Palo Cortado Sherry wines studied. (b) Higher alcohols, in mg/L, in the Oloroso and Palo Cortado Sherry wines studied.

(a)	Acetaldehyde	Diethyl-Acetal	Methanol	n-Propanol	Isobutanol
Oloroso Sherry Wine					
Y.P.F.W.	63.7 ± 10.0 ^a^	7.0 ± 1.0 ^a^	85.7 ± 4.7 ^a^	28.7 ± 5.5 ^a^	35.0 ± 4.0 ^a^
Añada	138.0 ± 6.0 ^b^	16.7 ± 1.5 ^b^	95.3 ± 2.1 ^b^	36.0 ± 1.7 ^b^	41.3 ± 2.5 ^b^
7th Cra.	159.7 ± 7.0 ^d,e^	25.3 ± 2.9 ^c^	101.0 ± 4.4 ^b^	40.7 ± 1.5 ^c^	47.3 ± 2.1 ^c^
6th Cra.	155.0 ± 6.6 ^c,d^	29.7 ± 2.5 ^d,e^	107.0 ± 4.6 ^c^	45.3 ± 2.5 ^d^	54.3 ± 2.3 ^d^
5th Cra.	152.0 ± 9.5 ^c^	31.3 ± 2.1 ^e,f^	111.0 ± 5.0 ^c,d^	47.7 ± 1.2 ^d,e^	58.0 ± 1.0 ^e^
4th Cra.	167.7 ± 4.0 ^f^	31.7 ± 3.2 ^e,f^	115.3 ± 3.2 ^d,e^	50.3 ± 2.5 ^e,f^	60.7 ± 1.5 ^e^
3rd Cra.	154.0 ± 6.6 ^c,d^	31.7 ± 1.5 ^e,f^	120.7 ± 4.5 ^e,f^	52.0 ± 2.6 ^f^	65.3 ± 2.1 ^f^
2nd Cra.	158.0 ± 4.6 ^c,d^	28.7 ± 1.5 ^d^	123.3 ± 4.0 ^f^	53.3 ± 1.5 ^f,g^	64.7 ± 3.1 ^f^
1st Cra.	165.7 ± 2.1 ^e,f^	33.0 ± 3.0 ^f^	133.3 ± 5.5 ^g^	55.3 ± 2.5 ^g^	71.7 ± 2.5 ^g^
Solera	155.7 ± 4.2 ^c,d^	29.3 ± 1.5 ^d,e^	144.0 ± 8.9 ^h^	61.0 ± 1.0 ^h^	77.3 ± 2.1 ^h^
Palo Cortado Sherry Wine					
Y.P.F.W.	72.3 ± 19.0 ^a^	7.0 ± 2.6 ^a^	65.7 ± 7.4 ^a^	30.0 ± 2.6 ^a^	37.7 ± 4.5 ^a^
S/T W. *	133.0 ± 11.4 ^b^	15.0 ± 1.0 ^b^	69.0 ± 4.4 ^a^	38.0 ± 3.6 ^b^	48.7 ± 2.1 ^b^
Añada	167.3 ± 5.5 ^d,e^	21.0 ± 2.0 ^c^	89.0 ± 2.0 ^b^	42.0 ± 3.0 ^c^	53.0 ± 2.0 ^c^
6th Cra.	162.3 ± 3.1 ^c,d,e^	20.3 ± 1.2 ^c^	94.7 ± 1.5 ^c^	45.7 ± 1.2 ^d^	55.7 ± 1.5 ^d^
5th Cra.	161.0 ± 3.0 ^c,d^	21.7 ± 1.5 ^c^	98.3 ± 2.1 ^c^	47.0 ± 1.0 ^d,e^	57.7 ± 1.5 ^d^
4th Cra.	157.3 ± 3.5 ^c^	27.7 ± 2.1 ^d^	102.7 ± 4.2 ^d^	49.0 ± 2.0 ^e,f^	60.3 ± 2.5 ^e^
3rd Cra.	167.7 ± 5.0 ^d,e^	28.7 ± 1.2 ^d,e^	108.0 ± 2.6 ^e^	51.3 ± 1.5 ^f^	62.0 ± 1.0 ^e,f^
2nd Cra.	171.0 ± 4.0 ^e^	31.0 ± 3.0 ^e^	126.3 ± 1.5 ^f^	59.7 ± 1.2 ^g^	64.3 ± 1.5 ^f^
1st Cra.	192.7 ± 5.1 ^f^	36.0 ± 2.6 ^f^	136.3 ± 1.5 ^g^	66.0 ± 2.0 ^h^	71.0 ± 2.0 ^g^
Solera	191.0 ± 4.4 ^f^	37.7 ± 2.1 ^f^	147.7 ± 3.5 ^h^	73.0 ± 2.0 ^i^	78.7 ± 1.5 ^h^
**(b)**	**2-Methyl-1-Butanol**	**3-Methyl-1-Butanol**		**Hexanol**	**2-Phenylethanol**
Oloroso Sherry Wine					
Y.P.F.W.	36.3 ± 4.2 ^a^	141.7 ± 12.7 ^a^		0.87 ± 0.15 ^a^	17.6 ± 11.3 ^a^
*Añada*	40.0 ± 3.0 ^b^	158.7 ± 8.0 ^b^		0.83 ± 0.15 ^a^	18.1 ± 3.3 ^a^
7th Cra.	44.3 ± 2.5 ^c^	170.7 ± 6.7 ^c^		0.83 ± 0.12 ^a^	20.2 ± 1.3 ^a,b^
6th Cra.	46.7 ± 1.5 ^c,d^	177.7 ± 3.5 ^c^		0.80 ± 0.10 ^a^	22.6 ± 0.8 ^b,c^
5th Cra.	50.3 ± 2.5 ^e^	188.3 ± 6.0 ^d^		0.93 ± 0.06 ^a,b^	23.6 ± 0.7 ^b,c,d^
4th Cra.	48.7 ± 2.1 ^d,e^	190.7 ± 2.5 ^d,e^		0.87 ± 0.12 ^a^	24.2 ± 0.6 ^b,c,d^
3rd Cra.	51.7 ± 2.5 ^e,f^	196.7 ± 3.8 ^e^		1.03 ± 0.15 ^b,c^	26.0 ± 0.7 ^c,d^
2nd Cra.	54.0 ± 3.0 ^f,g^	198.3 ± 5.5 ^e^		1.10 ± 0.10 ^c^	26.1 ± 0.5 ^c,d^
1st Cra.	56.3 ± 2.1 ^g^	208.0 ± 5.3 ^f^		1.03 ± 0.06 ^b,c^	27.9 ± 0.7 ^d^
*Solera*	60.3 ± 1.2 ^h^	220.3 ± 5.5 ^g^		1.10 ± 0.10 ^c^	27.7 ± 0.8 ^d^
Palo Cortado Sherry Wine					
Y.P.F.W.	40.0 ± 7.2 ^a^	154.3 ± 26.1 ^a^		1.17 ± 0.57 ^d^	16.9 ± 12.2 ^a^
S/T W. *	39.0 ± 4.4 ^a,b^	160.0 ± 13.1 ^a,b^		1.07 ± 0.21 ^c,d^	17.4 ± 1.6 ^a^
*Añada*	40.7 ± 1.2 ^a,b^	169.3 ± 3.2 ^b,c^		0.80 ± 0.10 ^a,b^	18.7 ± 1.4 ^a,b^
6th Cra.	42.7 ± 1.2 ^b,c^	172.3 ± 1.5 ^c^		0.83 ± 0.06 ^a,b,c^	21.1 ± 0.3 ^a,b,c^
5th Cra.	44.7 ± 2.1 ^c,d^	178.7 ± 4.0 ^c^		0.77 ± 0.06 ^a^	22.3 ± 1.0 ^b,c,d^
4th Cra.	46.3 ± 0.6 ^d^	192.7 ± 2.5 ^d^		0.87 ± 0.06 ^a,b,c^	25.5 ± 0.8 ^c,d,e^
3rd Cra.	47.7 ± 1.5 ^e,f^	196.7 ± 3.1 ^d^		0.87 ± 0.06 ^a,b,c^	26.9 ± 0.8 ^d,e,f^
2nd Cra.	50.0 ± 2.0 ^f,g^	203.0 ± 3.0 ^d^		0.90 ± 0.10 ^a,b,c^	28.0 ± 0.8 ^e,f^
1st Cra.	53.0 ± 2.0 ^g^	223.0 ± 4.4 ^e^		1.03 ± 0.06 ^b,c,d^	30.7 ± 0.2 ^f,g^
*Solera*	58.3 ± 1.5 ^h^	241.3 ± 7.0 ^f^		1.07 ± 0.06 ^c,d^	34.5 ± 0.9 ^g^

Mean values ± standard deviation (*n* = 4) are shown; ANOVA: for the same wine, different letters (a, b, c, d, e, f, g, h, i) indicate significant differences (*p* < 0.05). Y.P.F.W.: Young Palomino fortified wine; Cra: Criadera; S/T W. *: selected S/T wines for Palo Cortado; S/T: *Sobretabla*.

**Table 7:** (a) Ethyl acetate, ethyl esters of organic acids and ethyl esters of fatty acids in mg/L in the Oloroso and Palo Cortado Sherry wines studied. (b) Ethyl esters of fatty acids in mg/L in the Oloroso and Palo Cortado Sherry wines studied.

(a)	Ethyl Acetate	Ethyl Lactate	Diethyl Succinate	Ethyl Hexanoate	Ethyl Octanoate
Oloroso Sherry Wine					
Y.P.F.W.	45.7 ± 6.1 ^a^	17.07 ± 4.11 ^a^	0.93 ± 0.25 ^a^	0.27 ± 0.15	0.77 ± 0.15 ^a^
*Añada*	144.7 ± 8.5 ^b^	37.80 ± 0.79 ^b^	9.47 ± 0.78 ^b^	0.30 ± 0.10	1.07 ± 0.21 ^b^
7th Cra.	183.7 ± 4.2 ^c^	65.93 ± 0.99 ^c^	29.60 ± 0.79 ^c^	0.30 ± 0.10	1.27 ± 0.31 ^c^
6th Cra.	202.3 ± 11.2 ^d^	82.83 ± 1.45 ^d^	40.83 ± 1.21 ^d^	0.33 ± 0.12	1.37 ± 0.15 ^c^
5th Cra.	227.3 ± 3.5 ^e^	116.00 ± 6.24 ^e^	51.83 ± 1.23 ^e^	0.33 ± 0.06	1.57 ± 0.12 ^d^
4th Cra.	242.0 ± 6.1 ^f^	138.47 ± 1.27 ^f^	57.70 ± 0.92 ^f^	0.33 ± 0.06	1.63 ± 0.12 ^d,e^
3rd Cra.	256.7 ± 7.1 ^g^	150.87 ± 3.18 ^g^	65.67 ± 1.12 ^g^	0.33 ± 0.06	1.73 ± 0.06 ^d,e^
2nd Cra.	274.0 ± 3.0 ^h^	164.43 ± 0.93 ^h^	71.33 ± 0.40 ^h^	0.33 ± 0.06	1.80 ± 0.10 ^e,f^
1st Cra.	291.0 ± 2.0 ^i^	184.67 ± 1.55 ^i^	79.67 ± 0.74 ^i^	0.33 ± 0.06	1.97 ± 0.15 ^f,g^
*Solera*	323.7 ± 6.7 ^j^	203.65 ± 1.63 ^j^	89.67 ± 0.64 ^j^	0.37 ± 0.06	2.10 ± 0.10 ^g^
Palo Cortado Sherry Wine					
Y.P.F.W.	49.0 ± 6.1 ^a^	20.20 ± 3.97 ^a^	1.10 ± 0.35 ^a^	0.23 ± 0.15 ^a,b,c^	0.57 ± 0.25 ^a^
S/T W. *	74.7 ± 10.4 ^b^	34.80 ± 3.44 ^b^	5.77 ± 0.55 ^b^	0.23 ± 0.06 ^a,b,c^	0.73 ± 0.06 ^a^
*Añada*	113.0 ± 7.8 ^c^	55.63 ± 0.70 ^c^	25.83 ± 0.55 ^c^	0.20 ± 0.10 ^a,b^	3.87 ± 0.15 ^b^
6th Cra.	147.3 ± 4.0 ^d^	69.33 ± 0.40 ^d^	37.73 ± 0.85 ^d^	0.17 ± 0.06 ^a^	3.97 ± 0.21 ^b^
5th Cra.	161.3 ± 8.0 ^e^	87.73 ± 0.72 ^e^	48.43 ± 1.10 ^e^	0.20 ± 0.10 ^a,b^	4.20 ± 0.10 ^c^
4th Cra.	166.7 ± 5.0 ^e^	120.80 ± 1.37 ^f^	65.53 ± 1.20 ^f^	0.20 ± 0.10 ^a,b^	4.43 ± 0.12 ^d^
3rd Cra.	189.3 ± 3.5 ^f^	169.20 ± 1.28 ^g^	80.87 ± 1.05 ^g^	0.27 ± 0.06 ^a,b,c^	4.70 ± 0.10 ^e^
2nd Cra.	227.3 ± 3.8 ^g^	216.87 ± 4.46 ^h^	93.37 ± 1.50 ^h^	0.30 ± 0.10 ^b,c^	4.90 ± 0.10 ^f^
1st Cra.	275.7 ± 3.8 ^h^	279.70 ± 6.45 ^i^	126.00 ± 1.30 ^i^	0.33 ± 0.06 ^c^	5.20 ± 0.10 ^g^
*Solera*	325.3 ± 7.6 ^i^	370.17 ± 4.37 ^j^	171.63 ± 4.01 ^j^	0.33 ± 0.06 ^c^	5.30 ± 0.30 ^g^
**(b)**	**Ethyl** **Decanoate**	**Ethyl** **Dodecanoate**	**Ethyl** **Tetradecanoate**		**Ethyl** **Hexadecanoate**
Oloroso Sherry Wine					
Y.P.F.W.	0.20 ± 0.10 ^a^	0.20 ± 0.10 ^a,b^	0.07 ± 0.06		0.17 ± 0.06 ^a,b,c^
*Añada*	0.17 ± 0.06 ^a,b^	0.17 ± 0.06 ^a^	0.07 ± 0.06		0.20 ± 0.10 ^b,c^
7th Cra.	0.23 ± 0.06 ^a,b^	0.20 ± 0.10 ^a,b^	0.10 ± 0.10		0.23 ± 0.06 ^c^
6th Cra.	0.23 ± 0.12 ^a,b^	0.23 ± 0.06 ^a,b^	0.07 ± 0.12		0.23 ± 0.06 ^c^
5th Cra.	0.30 ± 0.10 ^b,c^	0.23 ± 0.06 ^a,b^	0.13 ± 0.06		0.20 ± 0.10 ^b,c^
4th Cra.	0.40 ± 0.10 ^c,d^	0.23 ± 0.06 ^a,b^	0.07 ± 0.06		0.17 ± 0.06 ^a,b,c^
3rd Cra.	0.43 ± 0.06 ^d^	0.27 ± 0.06 ^b^	0.10 ± 0.00		0.20 ± 0.10 ^b,c^
2nd Cra.	0.43 ± 0.15 ^d^	0.23 ± 0.06 ^a,b^	0.13 ± 0.06		0.17 ± 0.06 ^a,b,c^
1st Cra.	0.57 ± 0.15 ^e^	0.27 ± 0.06 ^b^	0.13 ± 0.06		0.10 ± 0.10 ^a^
*Solera*	0.70 ± 0.10 ^f^	0.23 ± 0.06 ^a,b^	0.13 ± 0.06		0.13 ± 0.12 ^a,b^
Palo Cortado Sherry Wine					
Y.P.F.W.	0.17 ± 0.12 ^a^	0.13 ± 0.06 ^a^	0.07 ± 0.06 ^a^		0.13 ± 0.06 ^a^
S/T W. *	0.18 ± 0.03 ^a^	0.17 ± 0.12 ^a,b^	0.07 ± 0.06 ^a^		0.13 ± 0.06 ^a^
*Añada*	0.33 ± 0.06 ^b,c^	0.13 ± 0.06 ^a^	0.07 ± 0.06 ^a^		0.13 ± 0.06 ^a^
6th Cra.	0.30 ± 0.10 ^b^	0.17 ± 0.06 ^a,b^	0.07 ± 0.06 ^a^		0.17 ± 0.06 ^a,b^
5th Cra.	0.30 ± 0.10 ^b^	0.20 ± 0.10 ^a,b^	0.07 ± 0.12 ^a^		0.17 ± 0.06 ^a,b^
4th Cra.	0.33 ± 0.06 ^b,c^	0.23 ± 0.06 ^b^	0.13 ± 0.06 ^a,b^		0.20 ± 0.10 ^a,b,c^
3rd Cra.	0.37 ± 0.06 ^b,c^	0.17 ± 0.06 ^a,b^	0.13 ± 0.06 ^a,b^		0.20 ± 0.10 ^a,b,c^
2nd Cra.	0.37 ± 0.06 ^b,c^	0.23 ± 0.06 ^b^	0.17 ± 0.06 ^b^		0.23 ± 0.06 ^b,c^
1st Cra.	0.40 ± 0.10 ^c,d^	0.17 ± 0.06 ^a,b^	0.20 ± 0.10 ^b^		0.23 ± 0.06 ^b,c^
*Solera*	0.47 ± 0.06 ^d^	0.23 ± 0.06 ^b^	0.17 ± 0.06 ^b^		0.27 ± 0.06 ^c^

Mean values ± standard deviation (*n* = 4) are shown; ANOVA: for the same wine, different letters (a, b, c, d, e, f, g, h, i, j) indicate significant differences (*p* < 0.05). Y.P.F.W.: Young Palomino fortified wine; Cra: Criadera; S/T W. *: selected S/T wines for Palo Cortado; S/T: *Sobretabla.*

**Table 8:** (a) Folin–Ciocalteau index FCI (milligrams of gallic acid per liter) and concentration of phenolic compounds (mg/L) in the Oloroso and Palo Cortado Sherry wines studied. (b) Concentration of phenolic compounds (mg/L) in the Oloroso and Palo Cortado Sherry wines studied. (c) Concentration of phenolic compounds (mg/L) and furfurals (mg/L) in the Oloroso and Palo Cortado Sherry wines studied.

(a)	FCI	Gallic Acid	p-Hydroxybenzoic Acid	Vanillic Acid	Syringic Acid	Protocatechuic Acid
Oloroso Sherry Wine						
Y.P.F.W.	290 ± 14 ^a^	5.08 ± 0.77 ^a^	0.33 ± 0.08 ^a^	0.13 ± 0.05 ^a^	0.40 ± 0.07 ^a^	0.34 ± 0.20 ^a^
*Añada*	362 ± 8 ^b^	6.53 ± 0.57 ^b^	0.35 ± 0.03 ^a^	0.65 ± 0.07 ^b^	0.94 ± 0.08 ^b^	2.81 ± 0.14 ^b^
7th Cra.	398 ± 16 ^c^	8.25 ± 0.21 ^c^	0.37 ± 0.04 ^a^	1.20 ± 0.08 ^c^	1.42 ± 0.06 ^c^	2.93 ± 0.11 ^b^
6th Cra.	440 ± 12 ^d^	8.65 ± 0.15 ^c,d^	0.77 ± 0.03 ^b^	1.42 ± 0.11 ^d^	2.26 ± 0.04 ^d^	4.07 ± 0.14 ^c^
5th Cra.	469 ± 17 ^e^	8.87 ± 0.10 ^d^	1.00 ± 0.07 ^c^	2.01 ± 0.13 ^e^	2.85 ± 0.08 ^e^	4.83 ± 0.22 ^d^
4th Cra.	532 ± 9 ^f^	10.02 ± 0.25 ^e^	1.07 ± 0.05 ^c^	2.55 ± 0.11 ^f^	3.61 ± 0.07 ^f^	5.35 ± 0.12 ^e^
3rd Cra.	582 ± 7 ^g^	11.20 ± 0.28 ^f^	1.22 ± 0.03 ^d^	3.34 ± 0.12 ^g^	4.92 ± 0.16 ^g^	6.94 ± 0.11 ^f^
2nd Cra.	630 ± 8 ^h^	13.06 ± 0.32 ^g^	1.31 ± 0.04 ^e^	4.03 ± 0.10 ^h^	5.76 ± 0.07 ^h^	8.51 ± 0.35 ^g^
1st Cra.	706 ± 10 ^i^	14.01 ± 0.22 ^h^	1.64 ± 0.10 ^f^	5.26 ± 0.07 ^i^	7.06 ± 0.09 ^i^	9.82 ± 0.18 ^h^
*Solera*	791 ± 7 ^j^	15.12 ± 0.21 ^i^	2.14 ± 0.12 ^g^	6.86 ± 0.18 ^j^	9.24 ± 0.08 ^j^	11.77 ± 0.44 ^i^
Palo Cortado Sherry Wine						
Y.P.F.W.	246 ± 13 ^a^	3.83 ± 0.31 ^a^	0.24 ± 0.10 ^a^	0.09 ± 0.02 ^a^	0.39 ± 0.14 ^a^	0.31 ± 0.12 ^a^
S/T W. *	274 ± 11 ^b^	4.67 ± 0.23 ^b^	0.39 ± 0.02 ^b^	0.33 ± 0.06 ^b^	0.47 ± 0.05 ^a^	0.52 ± 0.05 ^b^
*Añada*	332 ± 6 ^c^	7.75 ± 0.08 ^c^	0.62 ± 0.04 ^c^	0.82 ± 0.05 ^c^	1.30 ± 0.07 ^b^	3.05 ± 0.02 ^c^
6th Cra.	375 ± 9 ^d^	8.16 ± 0.15 ^d^	0.80 ± 0.03 ^d^	1.05 ± 0.07 ^d^	1.60 ± 0.06 ^c^	3.55 ± 0.07 ^d^
5th Cra.	407 ± 5 ^e^	8.68 ± 0.06 ^e^	0.91 ± 0.04 ^e^	1.33 ± 0.05 ^e^	1.83 ± 0.07 ^d^	4.03 ± 0.05 ^e^
4th Cra.	431 ± 6 ^f^	9.57 ± 0.09 ^f^	1.13 ± 0.08 ^f^	2.12 ± 0.12 ^f^	2.44 ± 0.06 ^e^	4.41 ± 0.19 ^f^
3rd Cra.	460 ± 8 ^g^	10.57 ± 0.13 ^g^	1.49 ± 0.11 ^g^	2.86 ± 0.10 ^g^	3.26 ± 0.08 ^f^	5.21 ± 0.15 ^g^
2nd Cra.	483 ± 6 ^h^	11.34 ± 0.22 ^h^	1.71 ± 0.08 ^h^	3.49 ± 0.09 ^h^	4.50 ± 0.16 ^g^	5.97 ± 0.12 ^h^
1st Cra.	511 ± 9 ^i^	12.79 ± 0.13 ^i^	1.81 ± 0.03 ^i^	4.55 ± 0.07 ^i^	5.91 ± 0.07 ^h^	6.30 ± 0.06 ^i^
*Solera*	598 ± 15 ^j^	14.26 ± 0.24 ^j^	1.92 ± 0.04 ^j^	6.25 ± 0.14 ^j^	7.66 ± 0.28 ^i^	7.13 ± 0.15 ^j^
**(b)**	**Caffeic Acid**	**p-Coumaric Acid**	**Ferulic Acid**	**p-Hydroxybenzal-** **dehyde**	**Vanillin**	**Syringal-** **dehyde**
Oloroso Sherry Wine						
Y.P.F.W.	3.87 ± 0.98 ^a^	0.55 ± 0.17 ^a^	0.41 ± 0.11 ^a^	0.16 ± 0.05 ^a^	0.28 ± 0.13 ^a^	0.62 ± 0.11 ^a^
*Añada*	4.80 ± 0.14 ^b^,^c^	1.21 ± 0.10 ^b^	0.47 ± 0.06 ^a^	0.56 ± 0.08 ^b^	0.43 ± 0.04 ^b^	1.12 ± 0.10 ^b^
7th Cra.	5.36 ± 0.06 ^d^,^e^	2.05 ± 0.14 ^c^	0.74 ± 0.06 ^b^	0.77 ± 0.05 ^c^	1.02 ± 0.09 ^c^	2.80 ± 0.04 ^c^
6th Cra.	5.67 ± 0.25 ^e^	2.73 ± 0.08 ^d^	0.94 ± 0.07 ^f^	1.07 ± 0.07 ^d^	1.52 ± 0.06 ^d^	3.97 ± 0.14 ^d^
5th Cra.	5.13 ± 0.10 ^c^,^d^	3.43 ± 0.08 ^e^	0.89 ± 0.05 ^e,f^	1.51 ± 0.09 ^e^	1.99 ± 0.07 ^e^	4.83 ± 0.09 ^e^
4th Cra.	4.41 ± 0.10 ^b^	4.07 ± 0.05 ^f^	0.77 ± 0.03 ^b,c^	1.97 ± 0.11 ^f^	2.12 ± 0.15 ^f^	5.82 ± 0.10 ^f^
3rd Cra.	3.79 ± 0.15 ^a^	4.74 ± 0.13 ^g^	0.85 ± 0.04 ^d,e^	2.19 ± 0.11 ^g^	2.62 ± 0.05 ^g^	6.54 ± 0.08 ^g^
2nd Cra.	3.23 ± 0.14 ^f^	5.47 ± 0.05 ^h^	0.76 ± 0.03 ^b^	2.46 ± 0.07 ^h^	2.89 ± 0.03 ^h^	7.53 ± 0.20 ^h^
1st Cra.	2.47 ± 0.06 ^g^	5.86 ± 0.06 ^i^	0.79 ± 0.05 ^b,c,d^	3.07 ± 0.13 ^i^	3.32 ± 0.05 ^i^	8.31 ± 0.12 ^i^
*Solera*	1.45 ± 0.09 ^h^	6.81 ± 0.06 ^j^	0.83 ± 0.04 ^c,d,e^	4.25 ± 0.07 ^j^	3.80 ± 0.11 ^j^	9.82 ± 0.32 ^j^
Palo Cortado Sherry Wine						
Y.P.F.W.	4.97 ± 0.71 ^a^	0.41 ± 0.10 ^a^	0.42 ± 0.13 ^a^	0.16 ± 0.08 ^a^	0.13 ± 0.04 ^a^	0.39 ± 0.10 ^a^
S/T W. *	4.25 ± 0.94 ^b^	0.44 ± 0.07 ^a^	0.44 ± 0.05 ^a^	0.28 ± 0.06 ^b^	0.15 ± 0.03 ^a^	0.43 ± 0.09 ^a^
*Añada*	3.89 ± 0.07 ^b^,^c^	1.50 ± 0.04 ^b^	0.51 ± 0.03 ^b,c^	0.60 ± 0.08 ^c^	0.73 ± 0.05 ^b^	2.23 ± 0.11 ^b^
6th Cra.	5.23 ± 0.07 ^a^	1.64 ± 0.03 ^c^	0.46 ± 0.03 ^a,b^	0.97 ± 0.05 ^d^	0.98 ± 0.03 ^c^	3.11 ± 0.09 ^c^
5th Cra.	5.14 ± 0.17 ^a^	1.89 ± 0.05 ^d^	0.44 ± 0.04 ^a^	1.22 ± 0.10 ^e^	1.23 ± 0.08 ^d^	3.99 ± 0.06 ^d^
4th Cra.	4.12 ± 0.03 ^b^	2.16 ± 0.04 ^e^	0.52 ± 0.03 ^b,c^	1.56 ± 0.08 ^f^	1.78 ± 0.09 ^e^	4.80 ± 0.08 ^e^
3rd Cra.	3.64 ± 0.08 ^c^	2.67 ± 0.13 ^f^	0.57 ± 0.02 ^c,d^	2.06 ± 0.11 ^g^	2.36 ± 0.08 ^f^	5.31 ± 0.08 ^f^
2nd Cra.	2.88 ± 0.04 ^d^	3.16 ± 0.08 ^g^	0.63 ± 0.03 ^d,e^	2.66 ± 0.08 ^h^	2.98 ± 0.04 ^g^	6.78 ± 0.05 ^g^
1st Cra.	2.30 ± 0.02 ^e^	4.03 ± 0.05 ^h^	0.69 ± 0.04 ^e^	3.38 ± 0.13 ^i^	3.27 ± 0.04 ^h^	8.10 ± 0.12 ^h^
*Solera*	1.49 ± 0.05 ^f^	5.66 ± 0.07 ^i^	0.85 ± 0.03 ^f^	4.35 ± 0.08 ^j^	4.04 ± 0.12 ^i^	10.59 ± 0.41 ^i^
**(c)**	**Trans-Caftaric Acid**	**Cis-** **p-Coutaric Acid**	**Trans-** **p-Coutaric Acid**	**Fertaric Acid**	**5-Hydroxymethylfurfural**	**Furfural**
Oloroso Sherry Wine						
Y.P.F.W.	35.53 ± 5.95 ^a^	8.11 ± 1.63 ^a^	11.16 ± 1.45 ^a^	7.61 ± 0.76 ^a^	1.83 ± 0.41 ^a^	0.38 ± 0.06 ^a^
*Añada*	29.93 ± 2.96 ^b^	5.83 ± 0.28 ^b^	9.35 ± 0.48 ^b^	6.88 ± 0.25 ^b^	7.32 ± 0.82 ^b^	5.17 ± 0.16 ^b^
7th Cra.	17.78 ± 0.91 ^c^	4.01 ± 0.09 ^c^	5.13 ± 0.11 ^c^	5.74 ± 0.14 ^c^	11.62 ± 0.24 ^c^	6.67 ± 0.09 ^c^
6th Cra.	15.34 ± 0.50 ^c^	3.11 ± 0.14 ^d^	4.66 ± 0.11 ^c,d^	5.07 ± 0.12 ^d^	16.54 ± 0.29 ^d^	7.64 ± 0.26 ^d^
5th Cra.	10.63 ± 0.86 ^d^	2.60 ± 0.20 ^d,e^	4.35 ± 0.11 ^d,e^	4.10 ± 0.10 ^e^	24.74 ± 0.56 ^e^	9.73 ± 0.13 ^e^
4th Cra.	7.57 ± 0.28 ^e^	2.11 ± 0.12 ^e,f^	4.18 ± 0.07 ^d,e,f^	3.50 ± 0.08 ^f^	33.32 ± 0.71 ^f^	11.37 ± 0.41 ^f^
3rd Cra.	5.17 ± 0.20 ^e,f^	1.69 ± 0.16 ^f,g^	3.97 ± 0.03 ^e,f^	2.69 ± 0.09 ^g^	43.60 ± 0.86 ^g^	15.52 ± 0.43 ^g^
2nd Cra.	3.70 ± 0.13 ^f,g^	1.36 ± 0.09 ^g^	3.91 ± 0.04 ^e,f^	2.27 ± 0.11 ^h^	57.61 ± 1.23 ^h^	19.14 ± 0.46 ^h^
1st Cra.	2.95 ± 0.18 ^f,g^	1.40 ± 0.13 ^g^	3.76 ± 0.11 ^f^	2.29 ± 0.10 ^h^	83.53 ± 1.49 ^i^	22.17 ± 0.42 ^i^
*Solera*	1.90 ± 0.24 ^h^	1.12 ± 0.13 ^g^	3.69 ± 0.14 ^f^	2.18 ± 0.19 ^h^	119.56 ± 3.99 ^j^	26.32 ± 1.55 ^j^
Palo Cortado Sherry Wine						
Y.P.F.W.	41.71 ± 13.83 ^a^	5.65 ± 0.52 ^a^	9.79 ± 2.13 ^a^	7.56 ± 1.19 ^a^	0.74 ± 0.09 ^a^	0.09 ± 0.04 ^a^
S/T W. *	28.65 ± 2.47 ^b^	5.04 ± 0.11 ^b^	7.24 ± 0.52 ^b^	5.00 ± 0.46 ^b^	1.29 ± 0.10 ^a^	0.19 ± 0.02 ^a^
*Añada*	20.88 ± 0.17 ^c^	4.12 ± 0.08 ^b^	6.62 ± 0.11 ^b^	4.74 ± 0.07 ^b^	8.86 ± 0.07 ^b^	3.88 ± 0.04 ^b^
6th Cra.	15.30 ± 0.29 ^d^	3.76 ± 0.12 ^c^	5.67 ± 0.06 ^c^	4.18 ± 0.06 ^c^	12.67 ± 0.22 ^c^	4.93 ± 0.16 ^c^
5th Cra.	11.21 ± 0.17 ^d,e^	3.03 ± 0.05 ^c,d^	4.94 ± 0.08 ^c,d^	3.69 ± 0.10 ^d^	16.20 ± 0.20 ^d^	6.08 ± 0.23 ^d^
4th Cra.	6.97 ± 0.07 ^e,f^	2.32 ± 0.06 ^d,e^	4.25 ± 0.08 ^d,e^	2.74 ± 0.07 ^e^	21.46 ± 0.38 ^e^	9.67 ± 0.16 ^e^
3rd Cra.	4.99 ± 0.09 ^f^	1.96 ± 0.08 ^e,f^	3.48 ± 0.04 ^e,f^	2.15 ± 0.08 ^f^	30.28 ± 0.16 ^f^	13.94 ± 0.32 ^f^
2nd Cra.	3.90 ± 0.07 ^f^	1.62 ± 0.05 ^f,g^	3.12 ± 0.11 ^f,g^	1.80 ± 0.06 ^f,g^	36.42 ± 0.49 ^g^	18.56 ± 0.17 ^g^
1st Cra.	2.93 ± 0.08 ^f^	1.50 ± 0.03 ^f,g^	2.96 ± 0.07 ^f,g^	1.56 ± 0.06 ^g,h^	41.29 ± 0.43 ^h^	22.21 ± 0.28 ^h^
*Solera*	1.86 ± 0.07 ^f^	1.32 ± 0.06 ^g^	2.35 ± 0.13 ^e^	1.12 ± 0.10 ^h^	56.14 ± 2.07 ^i^	27.01 ± 1.26 ^i^

Mean values ± standard deviation (*n* = 4) are shown; ANOVA: for the same wine, different letters (a, b, c, d, e, f, g, h, i, j) indicate significant differences (*p* < 0.05). Y.P.F.W.: Young Palomino fortified wine; Cra: Criadera; S/T W. *: selected S/T wines for Palo Cortado; S/T: *Sobretabla*.

**Table 9:** Absorbance values at 470 nm (a.u.) in the Oloroso and Palo Cortado Sherry wines.

	Oloroso Sherry		Palo Cortado Sherry
Y.P.F.W.	0.107 ± 0.017 ^a^	Y.P.F.W.	0.076 ± 0.003 ^a^
*Añada*	0.274 ± 0.011 ^b^	S/T W. *	0.119 ± 0.015 ^b^
7th Cra.	0.374 ± 0.007 ^c^	*Añada*	0.229 ± 0.009 ^c^
6th Cra.	0.445 ± 0.011 ^d^	6th Cra.	0.349 ± 0.012 ^d^
5th Cra.	0.509 ± 0.014 ^e^	5th Cra.	0.415 ± 0.008 ^e^
4th Cra.	0.628 ± 0.010 ^f^	4th Cra.	0.493 ± 0.011 ^f^
3rd Cra.	0.694 ± 0.007 ^g^	3rd Cra.	0.554 ± 0.011 ^g^
2nd Cra.	0.744 ± 0.015 ^h^	2nd Cra.	0.615 ± 0.012 ^h^
1st Cra.	0.869 ± 0.029 ^i^	1st Cra.	0.733 ± 0.009 ^i^
*Solera*	1.069 ± 0.020 ^j^	*Solera*	0.998 ± 0.014 ^j^

Mean values ± standard deviation (*n* = 4) are shown; ANOVA: for the same wine, different letters (a, b, c, d, e, f, g, h, i, j) indicate significant differences (*p* < 0.05). Y.P.F.W.: Young Palomino fortified wine; Cra: Criadera; S/T W. *: selected S/T wines for Palo Cortado; S/T: *Sobretabla*.

**Table 10:** Coefficients of principal components 1 (PC1) and 2 (PC2) of the variables that have shown the highest correlation (r > 0.12 in PC1 y > 0.24 in PC2).

Variables	PC1	PC2	Variables	PC1	PC2
Alcoholic strength	0.151542		2-Methyl-1-butanol	0.150198	
pH		0.324375	3-Methyl-1-butanol	0.151293	
Total Acidity	0.145974		2-phenylethanol	0.131638	
Volatile Acidity	0.150943		Ethyl lactate	0.147293	
Citric acid		−0.269015	Diethyl succinate	0.146096	
Lactic acid	0.153315		Ethyl octanoate		−0.356537
Malic acid		0.362702	Ethyl decanoate	0.130431	
Succinic acid	0.154153		FCI	0.148575	
Tartaric acid	−0.126789		Gallic acid	0.15686	
Glycerol	0.151733		Caffeic acid	−0.124957	
Total dry extract	0.156112		cis-p-Coutaric acid	−0.141108	
Total SO_2_	−0.140214		Fertaric acid	−0.142039	
Sulfates	0.14624		p-Coumaric acid	0.151824	
Calcium	0.128466		p-hydroxybenzoic acid	0.153595	
Potassium	0.132339		Protocatechuic acid	0.149546	
Reducing substances	0.147268		Syringic acid	0.156468	
Sugar-free extract	0.156677		trans-Caftaric acid	−0.139884	
A470	0.158087		trans-p-Coutaric acid	−0.132967	
Acetaldehyde		−0.249089	Vanillic acid	0.157568	
Diethyl-acetal	0.135741		p-Hydroxybenzaldehyde	0.157899	
Ethyl acetate	0.152624		Vanillin	0.158551	
Methanol	0.152534		Syringaldehyde	0.159106	
N-Propanol	0.149405		Furfural	0.157941	
Isobutanol	0.153078		5-Hydroxymethylfurfural	0.142912	

**Table 11:** Regression models (MLR) to estimate the ageing of Oloroso Sherry wines. O1: glycerol and organic acids model; O2: polyphenols model; O3: volatile compounds model; O4: “other variables” model; O5: overall model.

Model	Regression	R^2^ (Adjusted for DF)	*p* Value Model (95%)
O1	Average age (years) = −22.6605 + 0.000452103 × Tartaric acid + 0.0599505 × Citric acid + 0.00841926 × Succinic acid + 0.0164184 × Lactic acid	99.6548	0.0000
O2	Average age (years) = −67.6211 − 0.678351 × Diethyl − acetal + 0.360518 × Methanol + 0.307595 × 3-Methyl-1-butanol + 0.0972011 × Ethyl lactate − 22.9994 × Ethyl hexadecanoate	97.8803	0.0000
O3	Average age (years) = −2.58455 + 0.746821 × Gallic acid + 3.20304 × Vanillic acid + 2.74379 × Syringic acid − 6.72354 × Ferulic acid	99.8056	0.0000
O4	Average age (years) = −25.8569 + 13.324 × Volatile acidity + 0.00592469 × Potassium + 0.0506016 × FCI	99.6948	0.0000
**O5**	**Average age (years) = −18.2395 + 0.0319505 × Citric acid + 0.0115844 × Succinic acid + 0.00673568 × Lactic acid − 2.86101 ×** **Ferulic acid + 17.05 × Volatile acidity**	**99.9247**	**0.0000**

**Table 12:** Validation of the model O5 with eight of the analyzed Oloroso Sherry wine samples.

Sample	Average Age (Years)	Forecast Age (Years)	Standard Forecast Error	Absolute Error (Years)
8	4	4.01598	0.459898	−0.01598
13	9	8.6297	0.48663	0.3703
17	12	11.8062	0.452417	0.1938
22	18	18.7401	0.472403	−0.7401
27	24	23.7969	0.484317	0.2031
32	30	30.1405	0.473796	−0.1405
34	40	40.3205	0.463933	−0.3205
37	50	48.5899	0.532503	1.4101

Samples 8, 13, 17, 22, 27, 32, 34 and 37 are samples selected from the original data matrix to validate the proposed model and were not used in the MLR study.

**Table 13:** Regression models (MLR) to estimate the ageing of Palo Cortado Sherry wines. PC1: glycerol and organic acids; PC2: polyphenols model; PC3: volatile compounds model; PC4: “other variables” model; PC5: overall model.

Model	Regression	R^2^ (Adjusted for DF)	*p* Value Model (95%)
PC1	Average age (years) = 24.86 + 0.0018918 × Tartaric acid + 0.0160505 × Succinic acid + 0.0183707 × Lactic acid	99.5444	0.0000
PC2	Average age (years) = 6.22889 − 0.122881 × Diethyl − acetal − 0.103038 × Methanol + 0.0658476 × Ethyl lactate + 0.280439 × Diethyl succinate	99.7419	0.0000
PC3	Average age (years) = −6.9081 + 4.96422 × Vanillic acid − 1.70951 × protocatechuic acid + 0.943488 × Caffeic acid + 5.20505 × p-coumaric acid + 0.300888 × 5-Hydroxymethylfurfural	99.8840	0.0000
PC4	Average age (years) = −43.19 + 2.54966 × Total acidity + 23.9373 × Volatile acidity + 0.487494 × Total Dry Extract + 0.0496235 × FCI	99.8317	0.0000
**PC5**	**Average age (years) = −** **21.6426 + 0.00482137 ×** **Lactic acid + 2.79298 ×** **Total acidity + 9.20728 ×** **Volatile acidity + 2.90424 ×** **p-coumaric acid + 0.232434 ×** **5-Hydroxymethylfurfural**	**99.9150**	**0.0000**

**Table 14:** Validation of the model PC5 with nine of the analyzed Palo Cortado Sherry wine samples.

Sample	Average Age (Years)	Forecast Age (Years)	Standard Forecast Error	Absolute Error (Years)
74	1	0.552918	0.602941	0.44708
79	6	6.28852	0.578312	0.28852
83	10	9.38282	0.560119	0.61718
87	12	12.0188	0.563738	0.01880
91	18	18.2049	0.577721	0.20490
95	25	24.1583	0.587179	0.84170
99	30	31.1929	0.596577	1.19290
103	40	40.5200	0.556662	0.52000
108	60	59.7392	0.604779	0.26080

Samples 74, 79, 83, 87, 91, 95, 99, 103 and 108 are samples selected from the original data matrix to validate the proposed model and were not used in the MLR study.

**Table 15:** Tasting panel scores for the different descriptors of the 5 wines evaluated. Scores are expressed as mean value ± standard deviation of the panel. The *p* values resulting from the application of two-factor analysis of variance, taking the sample and the taster as the factors of variation, are included. Different superscripts in the scores indicate significant differences among the corresponding wines.

Sample	Aromatic Intensity	Nuts	Oak	Toasted/ Caramel	Citric	Lactic	Dryness	Equilibrium	Persistence
YPFW	1.6 ± 0.5 ^a^	1.0 ± 0.0 ^a^	1.0 ± 0.0 ^a^	1.0 ± 0.0 ^a^	1.3 ± 0.5 ^a^	1.4 ± 0.5 ^a^	1.4 ± 0.5 ^a^	2.1 ± 0.4 ^a^	1.6 ± 0.5 ^a^
O12YO	5.3 ± 0.8 ^c^	4.3 ± 0.8 ^b^	4.9 ± 0.7 ^c^	4.0 ± 0.8 ^b^	2.0 ± 0.6 ^a,b^	2.4 ± 0.5 ^b^	4.1 ± 0.7 ^b^	4.1 ± 0.7 ^b^	4.3 ± 0.5 ^b^
O+30YO	8.1 ± 0.9 ^d^	8.0 ± 0.8 ^c^	8.0 ± 0.8 ^d^	7.9 ± 0.7 ^d^	2.4 ± 0.5 ^b^	2.9 ± 0.7 ^b^	8.1 ± 0.7 ^d^	8.0 ± 0.8 ^c^	8.1 ± 0.9 ^c^
PC12YO	4.6 ± 1.0 ^b,c^	4.1 ± 0.9 ^b^	4.1 ± 0.7 ^b^	3.4 ± 0.5 ^b^	3.7 ± 0.8 ^c^	4.0 ± 0.8 ^c^	3.9 ± 0.7 ^b^	4.3 ± 0.5 ^b^	4.4 ± 0.8 ^b^
PC+30YO	8.0 ± 0.8 ^d^	7.7 ± 0.8 ^c^	7.6 ± 0.5 ^d^	6.0 ± 0.6 ^c^	7.1 ± 0.9 ^d^	7.0 ± 0.8 ^d^	7.1 ± 0.9 ^c^	8.1 ± 0.9 ^c^	8.0 ± 0.6 ^c^
*p*_sample_	0.000	0.000	0.000	0.000	0.000	0.000	0.000	0.000	0.000
*p*_taster_	0.987	0.883	0.910	0.944	0.725	0.792	0.895	0.848	0.911

Y.P.F.W.: Young Palomino fortified wine; O12YO: Oloroso 12 years old (5th Criadera); O+30YO: Oloroso over 30 years old (50% 1st Criadera + 50% 2nd Criadera); PC12YO: Palo Cortado 12 years old (5th Criadera); PC+30YO: Palo Cortado over 30 years old (50% 1st Criadera + 50% 2nd Criadera).
